# Organic nitrogen nutrition: LHT1.2 protein from hybrid aspen (*Populus tremula L. x tremuloides* Michx) is a functional amino acid transporter and a homolog of Arabidopsis LHT1

**DOI:** 10.1093/treephys/tpab029

**Published:** 2021-02-25

**Authors:** Regina Gratz, Iftikhar Ahmad, Henrik Svennerstam, Sandra Jämtgård, Jonathan Love, Mattias Holmlund, Rumen Ivanov, Ulrika Ganeteg

**Affiliations:** Umeå Plant Science Centre, Department of Forest Genetics and Plant Physiology, Swedish University of Agricultural Sciences, 90183 Umeå, Sweden; Department of Forest Ecology and Management, Swedish University of Agricultural Sciences, 90183 Umeå, Sweden; Umeå Plant Science Centre, Department of Forest Genetics and Plant Physiology, Swedish University of Agricultural Sciences, 90183 Umeå, Sweden; Umeå Plant Science Centre, Department of Forest Genetics and Plant Physiology, Swedish University of Agricultural Sciences, 90183 Umeå, Sweden; Department of Forest Ecology and Management, Swedish University of Agricultural Sciences, 90183 Umeå, Sweden; Umeå Plant Science Centre, Department of Forest Genetics and Plant Physiology, Swedish University of Agricultural Sciences, 90183 Umeå, Sweden; Umeå Plant Science Centre, Department of Forest Genetics and Plant Physiology, Swedish University of Agricultural Sciences, 90183 Umeå, Sweden; Institute of Botany, Heinrich Heine University, 40225 Düsseldorf, Germany; Umeå Plant Science Centre, Department of Forest Genetics and Plant Physiology, Swedish University of Agricultural Sciences, 90183 Umeå, Sweden

**Keywords:** amino acid transport, amino acid uptake, early senescence-like phenotype, hybrid aspen, lysine histidine transporter (LHT), nitrogen nutrition, organic nitrogen, *Populus tremula* L. x *tremuloides* Michx

## Abstract

The contribution of amino acids (AAs) to soil nitrogen (N) fluxes is higher than previously thought. The fact that AA uptake is pivotal for N nutrition in boreal ecosystems highlights plant AA transporters as key components of the N cycle. At the same time, very little is known about AA transport and respective transporters in trees. Tree genomes may contain 13 or more genes encoding the lysine histidine transporter (LHT) family proteins, and this complicates the study of their significance for tree N-use efficiency. With the strategy of obtaining a tool to study N-use efficiency, our aim was to identify and characterize a relevant AA transporter in hybrid aspen (*Populus tremula* L. x *tremuloides* Michx.). We identified PtrLHT1.2, the closest homolog of *Arabidopsis thaliana* (L.) Heynh AtLHT1, which is expressed in leaves, stems and roots. Complementation of a yeast AA uptake mutant verified the function of PtrLHT1.2 as an AA transporter. Furthermore, PtrLHT1.2 was able to fully complement the phenotypes of the Arabidopsis AA uptake mutant *lht1 aap5*, including early leaf senescence-like phenotype, reduced growth, decreased plant N levels and reduced root AA uptake. Amino acid uptake studies finally showed that PtrLHT1.2 is a high affinity transporter for neutral and acidic AAs. Thus, we identified a functional AtLHT1 homolog in hybrid aspen, which harbors the potential to enhance overall plant N levels and hence increase biomass production. This finding provides a valuable tool for N nutrition studies in trees and opens new avenues to optimizing tree N-use efficiency.

## Introduction

Nitrogen (N) is an essential element for life, and despite its high availability in the atmosphere, it represents a limiting factor for plant growth and development. This is due to the low rate of N fixation from the atmosphere in comparison with the high mobility of N, which accounts for high losses through leaching, volatilization or denitrification (Vitousek and Howarth 1991, LeBauer and Treseder 2008).

In order to cope with N deficiency, large amounts of commercial fertilizers are used in agriculture and forestry. Excessive use of inorganic N fertilizers has resulted in environmental pollution as well as adverse effects on human health (Gruber and Galloway 2008). Thus, decreasing the use of N fertilizers and increasing N-use efficiency without sacrificing biomass yield or product quality are major challenges for N nutrition research (McAllister et al. 2012).

Current models of the N cycle suggest that soil inorganic N, e.g.*,* nitrate and ammonium, are the main contributors to plant N. However, recent advances in soil biogeochemistry have shown that the contribution of amino acids (AAs) to soil N fluxes is much higher than previously thought, not only in poor soils, but also in soils of higher fertility (Inselsbacher and Näsholm 2012, Brackin et al. 2015, Oyewole et al. 2016, Ganeteg et al. 2017). In many boreal forest soils, AAs may account for up to 80% of the soil N composition (Inselsbacher and Näsholm 2012). This suggests that organic N, mainly AAs, plays a larger role in the N cycle than assumed. In a system dominated by organic N, uptake and allocation of AAs will be central to N nutrition, providing the plant with N (and carbon) to different tissues for growth, reproduction or storage. All of these processes are dependent on an intricate AA transport system, and AA transporters are thus key features of the N cycle. As building blocks for proteins and secondary metabolites, AAs are of central importance for plant growth and development and are considered major forms of N transport. Amino acids, taken up from the soil or synthesized from absorbed inorganic N in roots, are translocated from roots to shoots via the xylem. In contrast, AAs synthesized in source tissues are translocated to sink tissues via the phloem. Therefore, plants have a complex AA transport network, mediated by a group of AA transporters with different substrate specificities and tissue expression patterns (Rentsch et al. 2007, Tegeder 2014, Pratelli and Pilot 2014, Dinkeloo et al. 2018, Tegeder and Masclaux-Daubresse 2018).

In both the *Arabidopsis thaliana* (L.) Heynh and *Populus trichocarpa* (Torr. & Gray) genomes, more than 100 genes have been annotated as coding for known or putative AA transporters from different families (Schwacke et al. 2003, Tuskan et al. 2006). These transporters are classified in two main families: the amino acid transporter family (ATF) with 46 members and the amino acid-polyamine-choline family (APC) with 14 members in Arabidopsis (Rentsch et al. 2007). The ATF comprises six subfamilies: amino acid permeases (AAP), lysine histidine transporters (LHT), proline transporters (ProT), ϒ-aminobutyric acid transporters (GAT), auxin-resistant transporters (AUX), and aromatic and neutral amino acid transporters (ANT1-like) (Rentsch et al. 2007). Several determinants of N-use efficiency, such as uptake, cycling and remobilization of N are dependent on a well-orchestrated AA transport system (Good et al. 2007, Masclaux-Daubresse et al. 2010).

The 10 members of the Arabidopsis LHT (AtLHT) family (Rentsch et al. 2007) play diverse roles in different processes, such as root AA uptake (Hirner et al. 2006, Svennerstam et al. 2007, Svennerstam et al. 2011, Perchlik et al. 2014, Ganeteg et al. 2017), N cycling in mesophyll cells (Hirner et al. 2006), trichome development (Jakoby et al. 2008), pathogen attack (Liu et al. 2010, Elashry et al. 2013, Farjad et al. 2018, Yoo et al. 2020), import of AAs into the tapetum cells and transfer of organic N for pollen development (Lee and Tegeder 2004, Foster et al. 2008). One of the most thoroughly studied members is AtLHT1. Initial AA uptake studies in *Saccharomyces cerevisiae* overexpressing *AtLHT1* displayed a high affinity for L-Lys, L-His. Thus, this transporter was named after its substrate specificity for the basic AAs L-Lys and L-His (Chen and Bush 1997). However, subsequent studies in Arabidopsis revealed that AtLHT1 displayed high to medium affinity for most AAs, except for L-Lys and L-Arg (Hirner et al. 2006, Svennerstam et al. 2007). *AtLHT1* was found to be expressed in root epidermal and leaf mesophyll cells (Hirner et al. 2006). In addition, it was shown that AtLHT1 is involved in root uptake of acidic and neutral AAs at naturally occurring concentrations (Svennerstam et al. 2011) and from agricultural soil (Ganeteg et al. 2017). Interestingly, AtLHT1 is involved in responses to pathogen attack (Liu et al. 2010, Elashry et al. 2013, Farjad et al. 2018, Yoo et al. 2020) by potentially regulating AA availability. Furthermore, it was shown that AtLHT1 transports the ethylene precursor 1-aminocyclopropane-1-carboxylic acid (ACC) (Shin et al. 2014, Choi et al. 2019). The broad substrate affinity of AtLHT1 raised the question of this transporter would be suitable to shuttle bioactive components such as AA-based pesticides across the plasma membrane. By testing chlorantraniliprole-glycine-conjugates as well as glutamine-fipronil-conjugates it was confirmed that AtLHT1 actively takes up such conjugates, which opens up the possibility to use AtLHT1 as a novel delivery system for AA-based pesticide formulations (Chen et al. 2018, Jiang et al. 2018).

In a study about the molecular evolution of LHT transporters, it was shown that LHT homologs are present in charophytes, non-vascular land plants, non-seed vascular plants as well as in seed plants, and hence are broadly present throughout the plant kingdom (Tegeder and Ward 2012). In a recent study, OsLHT1 was identified to be a key transporter for root AA uptake, such as aspartate, but also to regulate AA allocation from root to shoot in rice (Guo et al. 2020). Disruption of *OsLHT1* caused severe growth defects and decreased yield (Wang et al. 2019). Guether et al. (2011) identified a gene encoding a high-affinity transporter similar to AtLHT1 in *Lotus japonicus*. LjLHT1.2 was shown to be involved in AA uptake at the fungus--root interface in mycorrhizal roots, which corresponds to recent expression studies performed on orchids (Zhao et al. 2014, Fochi et al. 2017). *Panax ginseng* PgLHT, similar to AtLHT1, was further shown to play a role in environmental stress responses (Zhang et al. 2013), displaying a broad regulatory function of LHT-type transporters. Even though several AA transporters have been characterized in Arabidopsis and other agriculturally valuable plants, not much is known about AA transporters in trees and their importance for N-use efficiency. A genome-wide survey of the AA transporter gene family in poplar showed that AA transporters might have key functions in various stress responses (Wu et al. 2015). Due to the potential involvement in different physiological and stress-related responses, further knowledge of AA transporters is crucially needed.

The main objective of this study was to increase our understanding about AA transport processes in trees through the identification and characterization of an AtLHT1 homolog in the model tree hybrid aspen (*Populus tremula* L. *x tremuloides* Michx.). This hybrid was chosen for its high potential in short-rotation plantation forestry, currently practiced in Nordic and Baltic countries, which can diminish human impact on the climate (Tullus et al. 2012). In boreal climates, *Populus tremula* L. *x tremuloides* Michx. displays one of the fastest growing rates and is currently of interest among breeders due to its productivity potential (Tullus et al. 2012). Another beneficial aspect is that hybrid aspen is easily propagated and genetically transformed (Yu et al. 2001, Sterky et al. 2004). Since hybrid aspen is not fully sequenced to date, we based our initial search for potential AtLHT1 homologs in hybrid aspen on the previously sequenced *P. trichocarpa* genome (Tuskan et al. 2006). Additionally, genetic tools are established for *P. trichocarpa*, which can be applied to hybrid aspen. We have established a system for heterologous expression of potential tree AA transporter homologs in Arabidopsis mutants, which display an overall reduced root AA uptake capacity as well as a disrupted N cycling in leaf mesophyll cells (Svennerstam et al. 2008, 2011). This approach enabled the identification and characterization of a functional *Populus* AA transporter, PtrLHT1.2. Upon expression in Arabidopsis mutants, PtrLHT1.2 rescued the early leaf-senescence phenotype and prompted increased root AA uptake. When grown on organic N as sole N source, plants displayed elevated N levels and thus increased biomass production. The finding that PtrLHT1.2 is an AA transporter and appears homologous to AtLHT1 extends the available toolbox for N nutrition studies in trees and opens new avenues to optimize tree N-use efficiency.

## Materials and methods

### Multiple sequence alignment between Arabidopsis and *P. trichocarpa* LHT members

The protein sequence of AtLHT1 (The Arabidopsis Information Resource [TAIR]) and the 13 poplar PtLHT members (Table S1 available as Supplementary data at *Tree Physiology* Online) (Wu et al. 2015) were aligned by using the multiple sequence alignment program Clustal Omega with default settings (https://www.ebi.ac.uk/Tools/msa/clustalo/) (Madeira et al. 2019). A Percent Identity Matrix was created subsequently.

### Motif discovery on Arabidopsis AtLHT1 and poplar LHT proteins

Conserved motifs within AtLHT1 and respective PtLHT protein sequences were identified with the help of the Multiple Em for Motif Elicitation tool (http://meme-suite.org/tools/meme, Version 5.1.1) (Bailey and Elkan 1994). Settings were adjusted as follows: motif site distribution: zero or one site per sequence, maximum number of motifs: 12, minimum/maximum motif width: 6/50, minimum/maximum sites per motif: 2/14.

### Prediction of transmembrane domains in LHT proteins

In order to find a suitable TMD prediction software, the ARAMEMNON 8 database for Arabidopsis integral membrane proteins has been used (Schwacke et al. 2003). The prediction for AtLHT1 suggested 11 TMDs when using the consensus prediction ConPred_v2 (Arai et al. 2004, Xia et al. 2004) as well as the extended consensus TM alpha helix prediction (AramTmMultiCon) provided by ARAMEMNON. The database can only predict TM properties of proteins from few plant species such as Arabidopsis, *Populus trichocarpa* or rice, though, and cannot predict TM properties in hybrid aspen proteins. In order to predict TM helices in all respective proteins addressed in this study, including PtrLHT1.2, the TMHMM Server (http://www.cbs.dtu.dk/services/TMHMM/, Version 2.0) (Krogh et al. 2001) was chosen for further analysis, as its prediction for AtLHT1 was in accordance with the ARAMEMNON predictions.

### Phylogenetic analysis of Arabidopsis and *P. trichocarpa* LHT members

The AA sequences of the 10 AtLHT (Tegeder and Ward 2012) an 13 PtLHT (Wu et al. 2015) family members were analyzed using the online tools at Phylogeny.fr (http://www.phylogeny.fr/index.cgi) (Dereeper et al. 2008, 2010) with the ‘advanced mode’ tool: (i) multiple alignment by MUSCLE (Edgar 2004), default settings; (ii) Gblocks treatment of alignments (Castresana 2000), default settings; and (iii) phylogenetic tree construction (Guindon and Gascuel 2003, Anisimova and Gascuel 2006, Chevenet et al. 2006) with 100 bootstrap replicates.

### Plant material

We denote a 16 h/8 h (light/dark) regime as long-day condition, while an 8/16 h regime corresponds to short day conditions. In both cases, a photosynthetic photon flux density of 150 μmol m^−2^ s^−1^ and a temperature of 22 °C/18 °C was ensured. An 18 h/6 h regime was maintained in the greenhouse.

To distinguish *Populus trichocarpa* from hybrid aspen, the following abbreviations are used in combination with respective gene codes: Pt: *P. trichocarpa*, Ptr: *Populus tremula* L. *x tremuloides* Michx. (hybrid aspen). Hybrid aspen wild-type (WT) (clone T89) was used in this study.

Different *A. thaliana* lines were used in this study: WT (ecotype Columbia, Col-0); *lht1–5* (SALK _115555, Svennerstam et al. 2007),* aap5–1* (SALK_041999, Svennerstam et al. 2008) as well as *lht1 aap5* double mutants (resulting from a cross of *lht1–5* with *aap5–1* mutants, Svennerstam et al. 2008). *lht aap5* double mutants, expressing *PtrLHT1.2* under control of the CaMV P35S promoter (*T1:3* and *T4:4*) were created in this study (description below).

### Hybrid aspen growth conditions

Tissue culture grown WT hybrid aspen were cultivated in Phytagel under long-day and sterile conditions. After 54 days, the plants were transferred either into liquid hydroponic solution or into soil.

#### Hydroponic cultivation

Prior to the hydroponic cultivation, a standard 50-ml falcon tube rack (4-Way Tube Rack, Fisher Scientific) was sawed into four individual pieces, each containing one well for a 50-ml falcon tube. In addition, liquid half-strength MS medium (Murashige and Skoog 1962) was prepared without addition of sucrose. Both was autoclaved and kept sterile.

The transfer of plantlets from Phytagel into the hydroponic system was carried out under sterile conditions: 200 ml of the MS liquid was added into closable jars, containing lids with ventilation strips. One piece of the sawed falcon tube rack was inserted into the jar. Afterwards, one plant was carefully inserted into the respective well. This construction allowed a stabilization of the fragile plantlets to obtain an upright positioning. The plants were then grown for four weeks under long-day conditions. Subsequently, plants were separated into leaves, stem and roots without a further tissue dissection. All samples were immediately frozen in liquid N and subsequently stored at −80 °C.

#### Growth in soil

For soil-grown poplar gene expression studies, plantlets with well-developed root systems were transferred to pots with soil:perlite (3:1) and grown in the greenhouse under long-day conditions for 46 days.

### Gene expression analysis of hybrid aspen tissues

Plants were then separated into roots (main and fine roots), stem (upper: 7 cm of upper stem, about 10 internodes below apex; and lower: 7 cm of lower stem, sampled above the first non-senescing leaf) and leaves (upper: top shoot including apex; middle: leaves sampled 10 internodes below apex; and lower: leaves attached to lower stem).

Extraction of mRNA was performed according to (Chang et al. 1993). After LiCl precipitation, the mRNA was purified using RNeasy mini kit (Qiagen) as described by the manufacturer. Genomic DNA was eliminated by Dnase I (Qiagen) digestion. cDNA was synthesized from 1 μg of RNA using the iScript cDNA synthesis Kit (Bio-Rad). Three technical replicates for each of the three biological replicates were run on a CFX96 real time PCR detection system using LightCycler SYBR Green Master Mix by Roche. The CT values from each technical replicate were then averaged for the corresponding biological replicate. Melting curve analysis confirmed the specificity of the reactions. Three different reference genes were tested (*EF1, UBQ, RP*) (Xu et al. 2011). All yielded similar results when used for normalization. Expression of the *Populus* ubiquitin extension protein gene *PtUBI3* was hence used to normalize the presented data. Primer pair sequences (PtUBI3_expression and PtLHT1.2_expression), and their efficiencies used for RT-qPCR are shown in Table S2 available as Supplementary data at *Tree Physiology* Online. Relative transcript levels for each sample were calculated for each biological replicate as: E^CT^_Reference_/E^CT^_Target_, where E is the obtained primer pair efficiency. Data are represented as mean values ± SE (*n* = 3).

### Cloning of PtrLHT1.2 from hybrid aspen

*PtrLHT1.2* was amplified from hybrid aspen cDNA using Platinum *Pfx* Polymerase (Invitrogen, Carlsbad, California, USA) with PtrLHT1.2_cloning primers listed in Table S2 available as Supplementary data at *Tree Physiology* Online. The blunt-end PCR product was cloned into the *pENTR/D-TOPO* vector, using the *pENTR* directional TOPO Cloning Kit (Thermo Fisher, Waltham, Massachusetts, USA). Forty colonies were tested by colony PCR to confirm correct orientation of the insert and 13 entry clones of *pENTR/D-TOPO::PtrLHT1.2* were sequenced. The alignment between PtrLHT1.2 with PtLHT1.2 and AtLHT1 was performed with Clustal Omega (https://www.ebi.ac.uk/Tools/msa/clustalo) (Madeira et al. 2019).

One positive clone was combined with the plant vector *pB7WG2D.1* (Karimi et al. 2002) using LR Clonase II (Invitrogen). Another colony PCR assured the correct orientation of the insert. This vector was then amplified in one-shot TOP10 chemically competent *E. coli* (Invitrogen) and prepared using QIAprep Spin Miniprep Kit (Qiagen, Hilden, Germany). *Agrobacterium tumefaciens GV3101::pMP90RK* (Koncz and Schell 1986) was transformed with the final construct (*pB7WG2D::35S::PtrLHT1.2*) using electroporation. Positive transformants were selected with 50 μg/ml spectinomycin and 25 μg/ml kanamycin.

### Tertiary protein structure prediction of PtrLHT1.2

The Protein Homology/analogY Recognition Engine (Phyre^2^) (http://www.sbg.bio.ic.ac.uk/∼phyre2/html/page.cgi?id=index , Version 2.0) in intensive mode was used to model the tertiary structure of PtrLHT1.2 (Kelley et al. 2015). The obtained PDB file was processed and visualized with EzMol (http://www.sbg.bio.ic.ac.uk/ezmol, Version 2.1) (Reynolds et al. 2018). The same PDB file was used to upload on the PoreWalker server (https://www.ebi.ac.uk/thornton-srv/software/PoreWalker) (Pellegrini-Calace et al. 2009).

### Protein localization by confocal microscopy

To generate a translational *PtrLHT1.2-GFP* fusion, the *PtrLHT1.2* sequence was amplified using PtrLHT1.2_fusion primers (Table S2 available as Supplementary data at *Tree Physiology* Online. The fragment was introduced into *pDONR207* (Invitrogen) by recombination and sequenced. In a second recombination step, the fragment was cloned into *pMDC83* binary vector (Curtis and Grossniklaus 2003), creating a *2x35Spro:PtrLHT1.2-GFP* expression cassette. The final vector was introduced into *Agrobacterium tumefaciens* C58C1 (pGV2260) strain. Constructs for the expression of OFP-HDEL (Batistic et al. 2008) and AHA1-mRFP (Caesar et al. 2011) markers were gifts from Prof. Dr J. Kudla and Prof. Dr K. Harter, respectively.

Transformation of tobacco was performed as in (Hötzer et al. 2012). Image acquisition was performed 48 h after infiltration and as described in (Ivanov et al. 2014). Plasmolysis was induced by 1 M mannitol, and images were taken after 7–15 min (Khan et al. 2019). ImageJ software (http://rsb.info.nih.gov/ij) was used for data evaluation. JaCoP v 2.0 plugin (Bolte and Cordelieres 2006) was used for calculating Pearson’s correlation coefficient and Manders’ coefficients for each PtrLHT1.2-marker combination. Costes’ automatic threshold was applied to ensure objective calculation of Manders’ coefficients. Line scan was performed using the RGB Profile Plot plugin. A minimum of six cells were used for the analysis of each combination.

### SDS--PAGE and immunoblotting

The procedure was performed as previously described (Ivanov et al. 2014). About 10 μg of protein were loaded per lane. The immunodetection was performed with anti-GFP-HRP conjugate antibody (Miltenyi Biotech, Bergisch Gladbach, Germany) at 1:1000 dilution.

### Yeast complementation assay

To prepare yeast expression constructs, the previously described *pENTR/D-TOPO::PtrLHT1.2* vector was used. As positive control, AtLHT1 was amplified with AtLHT1_cloning primers (Table S2 available as Supplementary data at *Tree Physiology* Online. The fragment was introduced into *pDONR207* (Invitrogen) by recombination and the resulting vector *pDONR207::AtLHT1* was sequenced. In a second recombination step, both genes were cloned into *pDRf1-GW* (Loque et al. 2007) using LR Clonase II (Invitrogen). As negative control *pDRf1-GW* without respective insert was chosen.

*Saccharomyces cerevisiae* strain 22574d (Jauniaux et al. 1987) was a gift from Prof. Dr B. André and was transformed with the three constructs according to the LiAc method (Ito et al. 1983). Positive clones were selected on medium, lacking uracil. Single colonies were selected for complementation studies. Complementation was performed on medium containing yeast N base without AAs and without ammonium sulfate, but which was supplemented with either 3 mM L-proline, L-citrulline or 3 mM GABA. As growth control, yeast was plated on 10 mM ammonium sulfate-containing medium. Non-supplemented medium served as negative control (Hirner et al. 2006). Pictures were taken after incubation at 30 °C for 10 days. The experiment was repeated three times and, one representative picture is shown.

### Generation of PtrLHT1.2 expressing lines

Seeds of *lht1 aap5* double mutants (Svennerstam et al. 2008) were stratified in 0.01% agarose at 4 °C for 2 days, sown on soil:perlite (3:1) and grown under long-day conditions until flowering. Mutants were transformed with *35S::PtrLHT1.2* (description above) using *Agrobacterium tumefaciens GV3101::pMP90RK*, according to the floral-dip method (Clough and Bent 1998). Lines were multiplied by selfing until homozygous. Two independent lines, *35S::PtrLHT1.2–1.3/lht1 aap5* (*T1:3*) and *35S::PtrLHT1.2.-4.4/lht1 aap5* (*T4:4*), were obtained for analysis. Positive transformants and lines were selected based on BASTA (Duchefa, Haarlem, The Netherlands) resistance and expression was confirmed by RT-qPCR analysis and uptake studies.

For phenotyping, seeds were sown on soil:perlite (3:1) and grown for 42 days under short-day conditions, or for 35 days under long-day conditions.

### Gene expression analysis of Arabidopsis tissues

Respective plant lines were grown on sterile vertical agar plates containing half-strength N-free Murashige and Skoog medium, 3 mM NO_3_^−^, 1% (w/v) agar and 0.5% (w/v) sucrose, buffered to pH 5.8 using 7.7 mM MES. Seeds were surface sterilized (Forsum et al. 2008), sown onto plates and stratified at 4 °C for 48 h. Plants were grown for 19 days under long-day conditions. Arabidopsis root mRNA was prepared using the RNeasy plant mini kit (Qiagen). Preparation of cDNA was performed as described before. Three technical replicates for each of the three biological replicates were run on a CFX96 real-time PCR detection system. The CT values from each technical replicate were then averaged for the corresponding biological replicate. Melting curve analysis confirmed the specificity of the reactions. The Arabidopsis Ubiquitin Ligase gene *AtUPL7* (At3g53090) was used to normalize the Arabidopsis RT-qPCR values (Czechowski et al. 2005). Sequences of used primers (AtUPL7_expression, AtLHT1_expression) are shown in Table S2 available as Supplementary data at *Tree Physiology* Online. The relative transcript levels for each sample were calculated for each biological replicate as: E^CT^_Reference_/E^CT^_Target_, where E is the obtained primer pair efficiency. Data are presented as mean values of three biological replicates (*n* = 3) ± SE. Different letters represent statistical significance (one-way ANOVA and Tukey post hoc test). The RT-qPCR product for *PtrLHT1.2* was sequenced to confirm amplification of the correct target gene.

### Nitrogen uptake analysis and biomass determination of PtrLHT1.2 overexpressing lines

Plants were grown under sterile and long-day conditions for 19 days on N-free MS medium, 0.5 mM L-Gln, 1% (w/v) agar and 0.5% (w/v) sucrose, buffered to pH 5.8 using 7.7 mM MES. The seedlings were dried at 60 °C and subsequently weighed. A minimum of four replicates were used for biomass determination. Afterwards, seedlings were homogenized for determination of total N content. Partially, samples were pooled to obtain a minimum of three biological replicates. The analysis was performed using an Elemental Analyzer—Isotope Ratio Mass Spectrometer (EA-IRMS) (EA: Flash EA 2000, IRMS: Delta V, both from Thermo Fisher Scientific) (Werner et al. 1999). Data presented are mean values ± SE (*P* < 0.05, one-way ANOVA with an additional Dunnett’s test, *n* = 3). Black stars indicate the comparison between *lht1*, *aap5* and *lht1 aap5* with the control Col-0, as their genetical background is Col-0. *T1:3* and *T4:4* were compared with *lht1 aap5*, as their genetical background is *lht1 aap5* (gray stars).

### ^14^C-amino acid uptake assay

Plant lines were grown on sterile vertical agar plates containing half-strength N-free MS medium, 3 mM NO_3_^−^, 1% (w/v) agar and 0.5% (w/v) sucrose, buffered to pH 5.8 using 7.7 mM MES. Seeds were surface-sterilized (Forsum et al. 2008), sown onto plates and stratified at 4 °C for 48 h. Plants were grown for 19 days under long-day conditions.

After removing plants from the agar, their roots were washed in 0.5 mM CaCl_2_ and gently blotted on tissue paper. Five biological replicates were prepared for each plant line. The AA uptake of Arabidopsis was assessed by submerging the roots of 19-day-old plants in 1 ml of 5, 10, 25 or 50 μM L-[U-^14^C]Gln (10 TBq mol^−1^) or L-[U-^14^C]Arg (12.1 TBq mol^−1^) solution (1.5 kBq ml^−1^) for 60 min. Subsequently, roots were washed three times in 0.5 mM CaCl_2_, separated from the shoot, dried at 40 °C and weighed. The tissue was rehydrated overnight in 200 μL of distilled water. Samples were digested in 1 ml Soluene 350 (Perkin Elmer, Waltham, Massachusetts, USA) in capped vials for 7 days. After the addition of 6 ml of scintillation cocktail (Hionic Fluor; Perkin Elmer), the samples were assayed for ^14^C in a Beckman LS6500 scintillation counter (Beckman Coulter, Solna, Sweden).

### Amino acid depletion assay

Plants were grown on sterile vertical agar plates containing half-strength N-free MS medium, 3 mM NO_3_^−^, 1% (w/v) agar and 0.5% (w/v) sucrose, buffered to pH 5.8 using 7.7 mM MES. Seeds were surface sterilized (Forsum et al. 2008), sown onto plates and stratified at 4 °C for 48 h. Plants were grown for 19 days under long-day conditions. The affinity of PtrLHT1.2 for a number of selected AAs was assessed by depletion, i.e.*,* the decline in concentration of each AA in the solution during the incubation is used to measure uptake. Nine plants from each line were divided into three biological replicates. The roots were rinsed in 0.5 mM CaCl_2_, blotted with tissue paper, placed in 2 ml of uptake solution and incubated in the climate-controlled chamber on a shaking table. The uptake solution contained 0.5 mM CaCl_2_ and the following compounds, each at a concentration of 10 μM and with pH adjusted to 5.8: L-Gln, L-Asn, L-Ala, L-Ser, L-Gly, L-Pro, L-Val, L-Glu, L-Asp and L-His. Samples of the uptake solution were taken after 1, 2 and 4 h. After the uptake period, roots were dried and weighed. The concentrations of the AAs in these samples were measured using the UPLC-AccQTag method (UPLC AA analysis system solution, www.waters.com), and data acquired from the 2 h samples were used to calculate AA uptake (μmol mg dry weight root^−1^ h^−1^). Data are presented as mean values of a minimum of three biological replicates (*n* = 3) ± SE. Different letters represent statistical significance (one-way ANOVA and Tukey post hoc test).

### Statistical analysis

Statistical analysis was performed using one-way ANOVA (*P* < 0.05), followed by either a Tukey’s or Dunnett’s post hoc test, respectively, both performed with OriginLab and JMP.

### Accession numbers

Arabidopsis sequence data from this article can be found in the EMBL/GenBank data libraries under accession numbers:

At5g40780 (*AtLHT1*); At1g24400 (*AtLHT2*); At1g61270 (*AtLHT3*); At1g47670 (*AtLHT4*); At1g67640 (*AtLHT5*); At3g01760 (*AtLHT6*); At4g35180 (*AtLHT7*); At1g71680 (*AtLHT8*); At1g25530 (*AtLHT9*); At1g48640 (*AtLHT10*); At2g18960 (*AtAHA1*); At3g53090 (*AtUPL7*).

Poplar sequence data from this article can be found in the *Populus* Genome Integrative Explorer database (PopGenIE v.3.0, www.popgenie.org)(Sjodin et al. 2009, Sundell et al. 2015) under accession numbers:

Potri.015G091600 (*PtLHT1*); Potri.001G335300.1 (*PtLHT1.2*); Potri.010G055800 (*PtLHT2*); Potri.010G128300 (*PtLHT3*); Potri.014G036500 (*PtLHT4*); Potri.008G179000 (*PtLHT5*); Potri.008G118000 (*PtLHT6*); Potri.009G140800 (*PtLHT7*); Potri.004G181200 (*PtLHT8*); Potri.002G012900 (*PtLHT10*); Potri.004G181100; Potri.001G335200; Potri.014G182400; Potri.014G115100 (*PtUBI.3*); PtrLHT1.2; NCBI: Accession Number: MW590808.

## Results

### Sequence analysis of the PtLHT family identified promising PtrLHT1.2 candidate in hybrid aspen

This study aims to identify a functional AA transporter in non-sequenced hybrid aspen, which is why a structured multi-step identification approach, consisting of six steps, has been applied (Figure S1 available as Supplementary data at *Tree Physiology* Online.

Step one consisted of a genome-wide analysis of the AA transporter gene family in *P. trichocarpa* (Wu et al. 2015), which revealed the presence of thirteen *PtLHT* gene models (Table S1 available as Supplementary data at *Tree Physiology* Online. In order to identify a potential AtLHT1 homolog, a multiple sequence alignment (Clustal Omega) between AtLHT1 protein and the 13 poplar candidates has been performed in a second step (Figure S2A available as Supplementary data at *Tree Physiology* Online. The three proteins with the highest identity to AtLHT1 were PtLHT1.2 (84.30%), Potri.001G335200 (81.61%) and PtLHT2 (77.45%) (Figure S2B available as Supplementary data at *Tree Physiology* Online, which thus represent good candidates for a functional AtLHT1 homolog. In order to identify and compare common motifs in between the protein sequences the Multiple Em for Motif Elicitation (MEME) tool was used (Figure S3 available as Supplementary data at *Tree Physiology* Online. Four different clusters could be formed, based on the identified motifs:

Cluster 1 comprising PtLHT1, PtLHT1.2, PtLHT2, PtLHT3, PtLHT5, PtLHT6 and Potri.001G335200. Cluster 2 encompasses PtLHT4, PtLHT7, PtLHT8 and PtLHT10. Potri.004G181100 (Cluster 3) and Potri.014G182400 (Cluster 4) do not exhibit any similarity with other motif arrangements and hence form individual clusters (Figure S3B and C available as Supplementary data at *Tree Physiology* Online. The identified clusters were slightly different compared with a previous motif analysis (Wu et al. 2015), which might be due to a different number of input sequences and hence comparisons. We additionally analyzed the motif arrangement for AtLHT1 and aimed to compare it to the different clusters to further narrow down the number of poplar candidates (step three). Interestingly, cluster 1 comprises all three potential AtLHT1 homolog candidates, namely PtLHT1.2, Potri.001G335200 and PtLHT2, and additionally displays the highest degree in conformity with the motif arrangement within AtLHT1 (Figure S3 available as Supplementary data at *Tree Physiology* Online. Hence, the number of interesting poplar candidates could not be further reduced in step three.

Step four, within the multi-step identification approach, consisted of an analysis of transmembrane arrangements within the proteins. We used the TMHMM Server v. 2.0 to predict 11 TMDs for AtLHT1, with an intracellular N-terminus and an extracellular C-terminus. This allowed us to show that only two out of the three putative poplar homologs exhibit the same membrane topology, which are PtLHT1.2 and PtLHT2 (Figure S4 available as Supplementary data at *Tree Physiology* Online.

**Figure 1.**
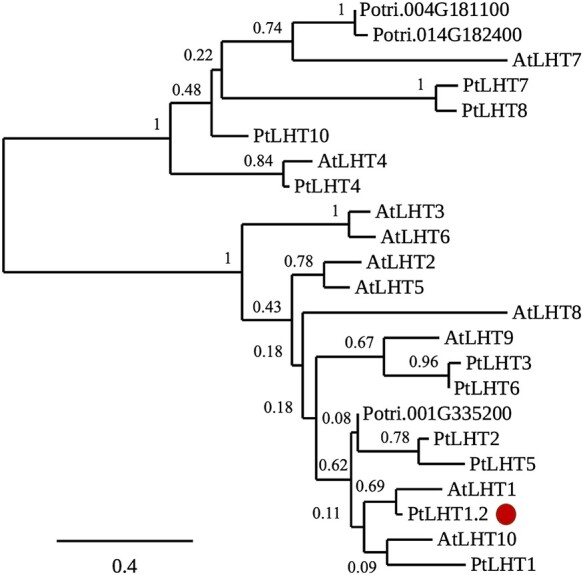
Phylogenetic relationship of the LHT family in Arabidopsis *thaliana* and *Populus trichocarpa*. The LHT proteins from both families group into two subfamilies. Cluster 1 contains six poplar proteins and AtLHT7 and AtLHT4. Cluster 2 contains seven poplar proteins and eight Arabidopsis LHT proteins. Central to the second cluster is PtLHT1.2 (highlighted in red), which is the closest relative to AtLHT1. The phylogenetic tree was constructed with the online tools at phylogeny.Fr based on the amino acid sequence of the respective candidate genes. Bootstrap values from 100 replicates are indicated at each node (whereas 1 indicates 100 out of 100 repetitions). The bar represents AA substitutions per position.

In order to identify the most promising, potential AA transporter candidate in hybrid poplar, we analyzed the phylogeny among all proteins (step five). The phylogenetic analysis revealed that the PtLHT family is divided into two subgroups (Figure 1): a smaller subgroup, with six poplar proteins and AtLHT7 and AtLHT4, and a larger subgroup, with seven poplar proteins. PtLHT1.2 was found to be most similar to AtLHT1, consistent with findings by Wu et al. (2015) and was hence chosen as promising target for further analysis.

Since the sequence analysis of transporter candidates in *P. trichocarpa* served as a tool to identify an AtLHT1 homolog in the yet not sequenced hybrid aspen tree *Populus tremula* L. *x tremuloides* Michx. (abbreviation Ptr), PtrLHT1.2 was chosen for further characterization and functional studies. As the final step six, an initial gene expression analysis was performed to analyze whether this gene is at all expressed in hybrid aspen (Table S2 available as Supplementary data at *Tree Physiology* Online, Figure S5 available as Supplementary data at *Tree Physiology* Online. Expression of the *Populus* ubiquitin extension protein gene *PtUBI3* was used for normalization and relative transcript levels were calculated as E^CT^_Reference_/E^CT^_Target_, where E is the obtained primer pair efficiency. The transcript abundance of *PtrLHT1.2* was confirmed in hybrid aspen. The highest expression of *PtrLHT1.2* was observed in leaves, but its presence was also shown in stem and root tissue of hydroponically grown hybrid aspen.

Thus, by applying a structured multi-step identification approach of predicted poplar AA transporters (Figure S1 available as Supplementary data at *Tree Physiology* Online, we could amplify the potential *AtLHT1* homolog *PtrLHT1.2* in hybrid aspen.

### PtrLHT1.2 is mainly expressed in leaf and root tissue and encodes a pore-forming transmembrane protein

**Figure 2.**
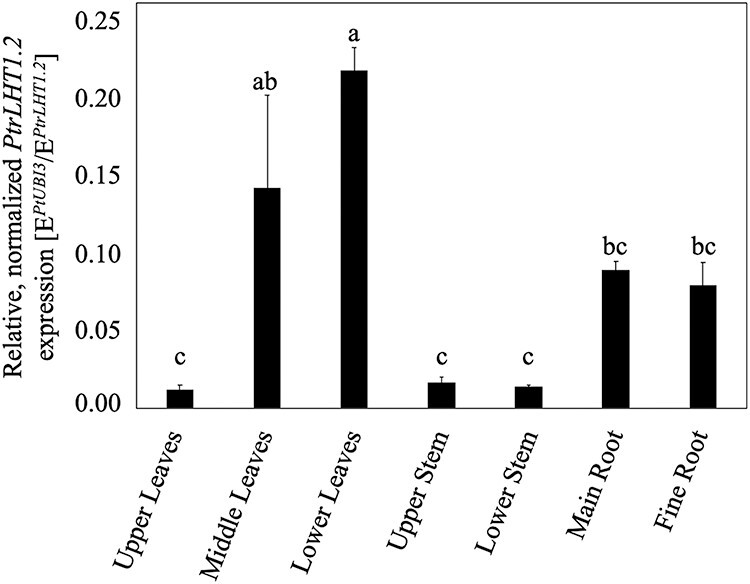
Differential transcript levels of *PtrLHT1.2* in poplar. Relative *PtrLHT1.2* transcript level analysis of in upper/middle and lower leaves, upper and lower stems and main and fine roots of 46-days-old hybrid aspen grown in soil. The *Populus* ubiquitin extension protein gene *PtUBI3* was used for normalization. Data are represented as mean values ± SE. Different letters indicate statistically significant differences (*P* < 0.05, one-way ANOVA and Tukey's post hoc test, *n* = 3).

To dissect the differential expression of *PtrLHT1.2* in more detail, mRNA was extracted from upper, middle, and lower leaves, upper and lower parts of the stem as well as main and fine roots of soil-grown young hybrid aspen. Similarly, *PtUBI3* transcript abundance was used for normalization and transcript levels were calculated according to E^CT^_Reference_/E^CT^_Target_. *PtrLHT1.2* was mainly expressed in middle and lower leaves and also in main and fine roots (Table S2 available as Supplementary data at *Tree Physiology* Online, Figure 2), which suggests a functional role in leaf and root tissue.

Subsequently, *PtrLHT1.2* was cloned. This approach yielded thirteen individual clones, all displaying the same sequence dissimilarities as compared with *PtLHT1.2*, with a discrepancy of 17 AA residues. This strongly suggests that a single *PtrLHT1.2* transcript was amplified using the selected primers. An alignment of the AA sequences of AtLHT1, PtLHT1.2 and PtrLHT1.2 is shown in Figure S6A available as Supplementary data at *Tree Physiology* Online. The sequence comparison revealed an identity of 96.2% and a similarity of 98.8% comparing both poplar proteins, which is in the same range as described (Sterky et al. 2004). Additionally, the resemblance of identified motifs within the three protein sequences exhibited a high degree of statistical significance (Figure S6B and C available as Supplementary data at *Tree Physiology* Online. Likewise, the TMD structure was identical in the newly identified hybrid aspen protein compared to AtLHT1 and PtLHT1.2, with 11 TMDs and an intercellular N-terminus and an extracellular C-terminus (Figure S6D available as Supplementary data at *Tree Physiology* Online. The presence of TMDs suggests, that PtrLHT1.2 is a transmembrane protein with a potential role as transporter.

In order to test the hypothesis that PtrLHT1.2 has structures needed for solute transport across a membrane, such as AAs, we investigated whether PtrLHT1.2 possesses a cavity, such as a pore, spanning the whole protein. Hence its respective protein structure was modeled based on protein homology/analogy recognition (Phyre^2^, EzMol) (Figure S7A available as Supplementary data at *Tree Physiology* Online. Based on this structure, it was tested whether a pore could be identified (Pore Walker) (Figure S7B--D available as Supplementary data at *Tree Physiology* Online. A vertical section along the pore axis was generated (Figure S7B available as Supplementary data at *Tree Physiology* Online whereas red spheres represent the pore centers at 1 Ångström (Å) steps. A horizontal section of the pore (height is marked in magenta, Figure S7C available as Supplementary data at *Tree Physiology* Online is presented as 2 Å slices, viewed from the top (top picture) and the bottom (lower picture) of the pore (Figure S7D available as Supplementary data at *Tree Physiology* Online. The finding that pore-forming AAs are present in PtrLHT1.2 supports the hypothesis, which PtrLHT1.2 might function as an AA transporter, similar to AtLHT1.

### Subcellular localization of PtrLHT1.2-GFP confirmed its presence at the plasma membrane

**Figure 3.**
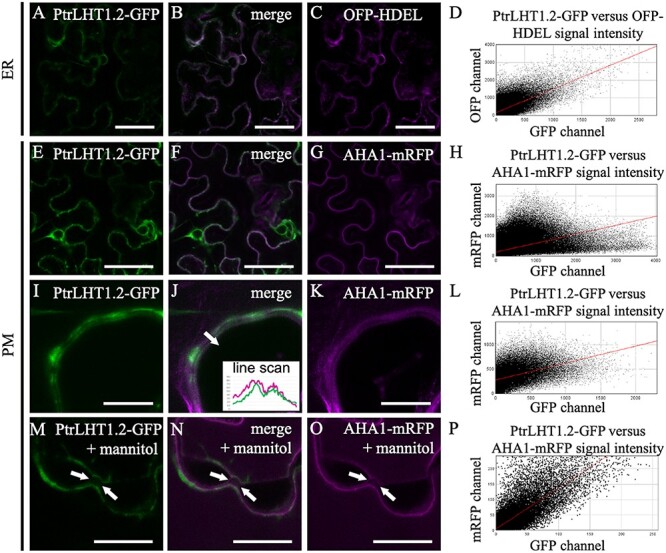
Localization of PtrLHT1.2-GFP in tobacco leaf epidermis. (A--C) co-localization between PtrLHT1.2-GFP and OFP-HDEL. GFP channel (A) is colored in green, while the OFP channel (C) is in magenta. Size bars represent 50 μm. (D) Scatter plot of PtrLHT1.2-GFP versus OFP-HDEL pixel intensities. (E--G) co-localization between PtrLHT1.2-GFP and AHA1-mRFP. GFP channel (E) is colored in green, while the RFP channel (G) is in magenta. Size bars represent 50 μm. (H) Scatter plot of PtrLHT1.2-GFP versus AHA1-mRFP pixel intensities. (I--K) co-localization between PtrLHT1.2-GFP and AHA1-mRFP in the PM region. GFP channel (I) is colored in green, while the RFP channel (K) is in magenta. The insert in (J) represents a line scan through the PM. The scanned region is indicated by a white arrow. Size bars represent 5 μm. (L) Scatter plot of PtrLHT1.2-GFP versus AHA1-mRFP pixel intensities. (M--O) co-localization between PtrLHT1.2-GFP and AHA1-mRFP in the PM region in mannitol-plasmolyzed cells. Plasmolysis is indicated by a white arrow. GFP channel (M) is colored in green, while the RFP channel (O) is in magenta. Size bars represent 50 μm. (P) Scatter plot of PtrLHT1.2-GFP versus AHA1-mRFP pixel intensities. Displayed are representative images from at least six cells analyzed.

In order to investigate PtrLHT1.2 localization, a PtrLHT1-GFP fusion was expressed in tobacco (*Nicotiana benthamiana*) leaf epidermis cells. Localization in different cellular compartments was found, which is why PtrLHT1-GFP was co-expressed with either the plasma membrane (PM) marker AHA1-mRFP (Caesar et al. 2011) or the endoplasmic reticulum (ER) marker peptide OFP-HDEL (Batistic et al. 2008). PtrLHT1.2-GFP showed strong ER localization (Figure 3A--C). Scatterplot of signal intensity indicated a good correlation between PtrLHT1.2-GFP and OFP-HDEL signals (Figure 3D). Over 63% of the GFP signal overlapped with OFP (Table S3 available as Supplementary data at *Tree Physiology* Online. Co-localization with the PM marker was weaker, with 22% of PtrLHT1.2-GFP present in AHA1-mRFP-positive structures (Table S3 available as Supplementary data at *Tree Physiology* Online and Figure 3E--H). A closer signal inspection of the PM region only (Figure 3I--K) as well as mannitol-induced plasmolysis (Figure 3M--O) showed better correlation in signal intensity (Figure 3L and P) and a 31 and 34% overlap between PtrLHT1.2-GFP and the marker (Table S3 available as Supplementary data at *Tree Physiology* Online. In order to ensure that the observed localization is due to the full-length PtrLHT1.2-GFP only, total protein was extracted, separated on a 12% SDS PAGE, and analyzed by immunoblot for potential degradation products (Figure S8 available as Supplementary data at *Tree Physiology* Online. A predominant band with an approximate molecular mass of 75 kDa was visible, corresponding to the full-length PtrLHT1.2-GFP. A weak band around 27 kDa was additionally detected, presumably corresponding to free GFP in sufficiently low amounts so as not to significantly influence the results of the co-localization experiments. Interestingly, a high molecular mass smear was observed, indicating potential post-translational modifications of the protein.

These results suggest that while large amounts of PtrLHT1.2-GFP are located in the ER, the protein has a significant presence at the PM. This is consistent with its potential function as a plasma membrane transport protein.

### Expression of PtrLHT1.2 in a yeast AA uptake mutant rescues growth on AAs

*Saccharomyces cerevisiae* strain 22574d is deficient in citrulline, proline and γ-aminobutyric acid (GABA) transport (Jauniaux et al. 1987) and was used for heterologous expression of *PtrLHT1.2.* As shown in Figure S9 available as Supplementary data at *Tree Physiology* Online, the presence of PtrLHT1.2 mediated growth of the yeast mutant on either 3 mM L-proline (Figure S9D available as Supplementary data at *Tree Physiology* Online, L-citrulline (Figure S9E available as Supplementary data at *Tree Physiology* Online or GABA (Figure S9F available as Supplementary data at *Tree Physiology* Online as sole N source respectively, and hence complemented the yeast (AA) uptake phenotype. Similar growth was detected when AtLHT1 was expressed as positive control (Hirner et al. 2006) and an expected, but severely reduced, background growth was visible upon expression of the empty vector only (negative control).

The ability of PtrLHT1.2 to mediate growth of an AA uptake mutant on e.g., L-proline as sole N source confirms that the protein is a functional AA transporter.

### Expression of PtrLHT1.2 rescued the early leaf-senescence and growth phenotype of the Arabidopsis lht1 aap5 double mutant

We developed an in planta test system, based on the Arabidopsis AA transporter *lht1 aap5* double mutant in order to characterize putative AA transporters with unknown substrate specificities. The AA uptake profiles of AtLHT1 and AtAAP5 are complementary: while AtLHT1 is mainly involved in uptake of acidic and neutral AAs, AtAAP5 targets uptake of basic AAs, respectively. Mutation of *aap5* has not revealed any other phenotypes than impaired uptake of basic AAs. The *lht1* mutation causes an impairment in leaf mesophyll N cycling, resulting in an early senescence-like phenotype (Hirner et al. 2006), and is responsible for reduced plant biomass when grown on L-Gln (Svennerstam et al. 2007, 2008). Overall, the *lht1 aap5* double mutant displays a severely decreased AA root uptake capacity, whereas only approximately 20% of WT AA uptake capacity is retained (Svennerstam et al. 2008, 2011). Thus, the *lht1 aap5* double mutant provides an excellent genetic background to assess whether expression of a presumable AA transporter rescues (i) the root uptake phenotype regarding both, acidic and neutral as well as basic AAs, (ii) the early senescence-like phenotype and (iii) the reduced plant biomass.

Hence, two individual Arabidopsis lines expressing *PtrLHT1.2* in an *lht1 aap5* double mutant background were generated under the control of the CaMV-35S promoter. By comparing the relative, endogenous *AtLHT1* expression in WT with the expression of *PtrLHT1.2* in the double mutants, we could confirm the generation of two overexpression lines: Line *T1:3* displayed an approximately two-fold and line *T4:4* an approximately 10-fold higher *PtrLHT1.2* expression as *AtLHT1* in WT (Table S2 and Figure S10 available as Supplementary data at *Tree Physiology* Online. No *PtrLHT1.2* transcript was detected in WT and the *lht1 aap5* double mutants. The Arabidopsis ubiquitin ligase gene *AtUPL7* (At3g53090) was used for normalization and relative transcript levels were calculated according to E^CT^_Reference_/E^CT^_Target_.

**Figure 4.**
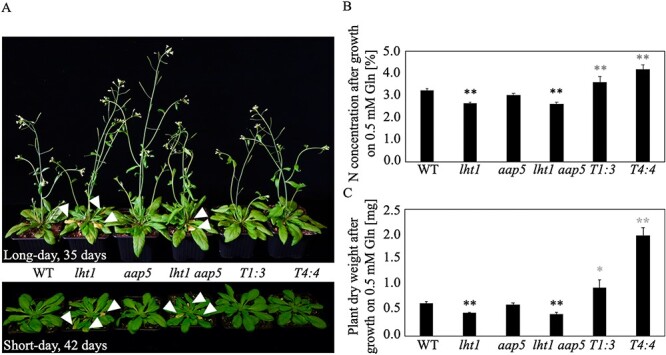
Heterologous *PtrLHT1.2* expression rescues AA transporter mutant phenotype. Characterization of Arabidopsis WT, *lht1, aap5, lht1 aap5* double mutants and two individual lines of *lht1 aap5* double mutant plants overexpressing *PtrLHT1.2* (*T1:3* and *T4:4*). (A) Plants were grown for either 35 days under a long-day regime (16 h/8 h day/night) (upper panel), or for 42 days under short-day regime (8 h/16 h day/night) (lower panel) at 22 °C with a photosynthetic photon flux density of 120 μ mol m^−2^ s^−1^. White arrow heads highlight the early-senescence phenotype. (B) Total plant N content (in %) is displayed after growth on 0.5 mM L-Gln as sole N source for 19 days under a long-day regime. Data are represented as mean values of a minimum of three biological replicates ± SE. Stars indicate statistically significant differences (*P* < 0.05, one-way ANOVA with an additional Dunnett’s test, *n* = 3). Black stars indicate the comparison between *lht1*, *aap5* and *lht1 aap5* with the control Col-0, as their genetical background is Col-0. *T1:3* and *T4:4* were compared with *lht1 aap5*, as their genetical background is *lht1 aap5* (gray stars). (C) Seedlings were dried and weighted after growth on 0.5 mM L-Gln as sole N source for 19 days under a long-day regime. Data are represented as mean values of a minimum of four biological replicates ± SE. Stars indicate statistically significant differences (*P* < 0.05, one-way ANOVA with an additional Dunnett’s test, *n* = 3). Black stars indicate the comparison between *lht1*, *aap5* and *lht1 aap5* with the control Col-0, as their genetical background is Col-0. *T1:3* and *T4:4* were compared with *lht1 aap5*, as their genetical background is *lht1 aap5* (gray stars).

Both *PtrLHT1.2-*expressing lines showed no signs of early senescence as opposed to the *lht1* single as well as the *lht1 aap5* double mutants, neither after 42 days under short-day conditions nor after 35 days under long-day conditions (Figure 4A).

In addition, the N concentration of 19-day-old seedlings was compared after growth on 0.5 mM L-Gln as sole N source. Both *PtrLHT1.2* expressing lines could revert the N deficiency, visible in the *lht1 aap5* mutant, and showed increased N levels. In order to demonstrate that reduced N levels are due to the mutation of the *LHT1* gene, we included the *lht1* as well as the *aap5* single mutant lines as controls (Figure 4B). The increase in plant N observed in overexpressing lines additionally led to a significant increase in plant biomass in those lines, even above WT levels, when grown on L-Gln. Individual *lht1* and *aap5* mutants were included as well to demonstrate that the reduced biomass accumulation is due to the disruption of the *LHT1* gene (Figure 4C).

Thus, heterologous expression of *PtrLHT1.2* rescued the *lht1*-caused early senescence-like and growth phenotype of the double mutant, suggesting it to have similar physiological characteristics as AtLHT1.

### Amino acid uptake capacity confirms role of PtrLHT1.2 as amino acid transporter

In planta, AtLHT1 mediates mainly the uptake of neutral and acidic AAs such as L-Gln while AtAAP5 targets the basic AAs L-Arg and L-Lys (Hirner et al. 2006, Svennerstam et al. 2007, 2008, 2011, Ganeteg et al. 2017). As PtrLHT1.2 conferred AA uptake in yeast AA uptake mutants, the protein’s ability to take up AAs and its substrate specificity in planta were tested. Thus, the double mutant was of benefit in order to create a situation in which the plant has an overall reduced AA uptake covering not just acidic and neutral AAs, but also basic ones.

**Figure 5.**
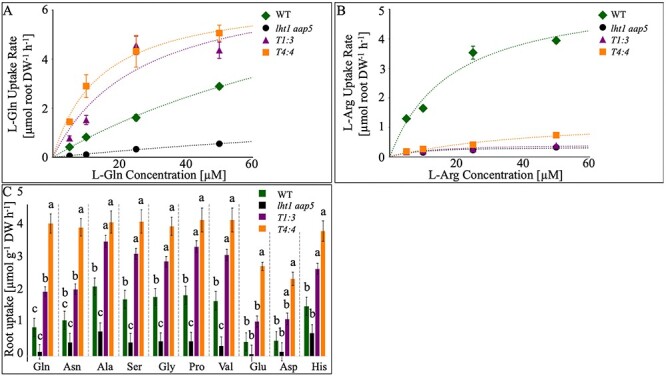
PtrLHT1.2 is a functional root amino acid transporter. (A--B): AA uptake in WT (green rhomb), *lht1 aap5* double mutants (black circle) and *PtrLHT1.2*-expressing *lht1 aap5* double mutants (T1:3 purple triangle and T4:4 orange square). Uptake of ^14^C-labeled L-Gln (A) and L-Arg (B) was analyzed by immersing roots of intact, axenically grown 19 days old plants in 5, 10, 25, and 50 μM of the respective AA. Amino acid uptake was calculated from the sum of ^14^C in shoots and roots and expressed per unit root dry mass. Each data point represents mean values of five biological replicates ± SE (n = 5). The curves show the Michaelis–Menten fit of the data. (C) Root AA uptake of 19-day-old WT (black bars), *lht1 aap5* double mutants (white bars) and two individual lines of *lht1 aap5* double mutant plants expressing *PtrLHT1.2* (T1:3; light gray bars and T4:4; dark gray bars). The roots were incubated in a mixture of different AAs, each at a concentration of 10 μM each. The uptake of each AA from the solution was calculated as μmol mg dry weight root-1 h-1. Bars represent mean values of a minimum of three biological replicates (*n* = 3) ± SE. Different letters represent statistical significance (one-way ANOVA and Tukey post hoc test).

Arabidopsis roots were submerged in different concentrations of ^14^C-labeled L-Gln and tested for their uptake capacity (Figure 5). The *PtrLHT1.2*-expressing *T4:4* and *T3:1* lines displayed the highest L-^14^C-Gln uptake rate, followed by WT. To test the ability of *PtrLHT1.2* to coordinate uptake of basic AAs, this experiment has been repeated with ^14^C-labeled L-Arg. Only WT displayed high affinity for L-^14^C-Arg, suggesting that similar to AtLHT1, PtrLHT1.2 only poorly transports the basic AA L-Arg (Figure 5A and B).

As overall confirmation that AtLHT1 and PtrLHT1.2 show similar uptake affinity profiles, we analyzed root AA uptake capacity with a broad spectrum of neutral and acidic AAs. Arabidopsis roots were incubated in a mixture of different AAs, such as L-Gln, L-Asn, L-Ala, L-Ser, L-Gly, L-Pro, L-Val, L-Glu, L-Asp and L-His (Svennerstam et al. 2008) (Figure 5C). The uptake of each AA was measured by its respective depletion from the mixture, reflecting the relative net uptake. The overexpressing lines consistently showed an increased uptake of the respective AAs compared with WT and the double mutant. Line *T4:4* yielded a higher uptake, which is consistent with the higher gene expression of *PtrLHT1.2* in that line (Figure S10 available as Supplementary data at *Tree Physiology* Online.

This demonstrates that PtrLHT1.2 not only relieves the AA uptake phenotype caused by the *lht1* mutation but is also capable of increasing the overall uptake of neutral and acidic AAs upon overexpression. An increased uptake of AAs hence leads to elevated N levels in plant tissue and thus increased biomass production. In summary, we could identify PtrLHT1.2 to be an AA transporter, which might be a functional hybrid aspen homolog of AtLHT1.

## Discussion

### A multi-step identification approach revealed a PtrLHT candidate gene

A phylogenetic analysis of AAP and LHT proteins revealed that both transporter families belong to two distinct groups in plants (Tegeder and Ward 2012). In contrast to AAP proteins, LHT proteins evolved prior to the existence of land plants, as they have been found in green algae and are therefore evolutionarily older than AAPs. Hence, LHT proteins seem to hold important functions from the onset of plant evolution. No tree species were included in the phylogenetic analysis by Tegeder and Ward (2012), and in general, very little is known about tree AA transport and their respective importance for N-use efficiency in forest ecosystems. Couturier et al. showed that poplar PtAAP11 holds important roles in xylem differentiation by supplying proline to xylem cell wall formation (Couturier et al. 2010*a*). Similarly, PtCAT11 was identified as an L-Gln transporter involved in source to sink transport during senescence (Couturier et al. 2010*b*). To our knowledge, LHT homologs from tree species have not yet been functionally characterized in detail. Thus, we aimed for a molecular understanding of LHT transporters in the economically important hybrid aspen (*Populus tremula* L. *x tremuloides* Michx.). As no genome sequence is yet available for hybrid aspen, we made use of the closely related *P. trichocarpa*. Coding sequences for both tree species are predicted to be highly similar (Sterky et al. 2004). Through a combined effort consisting of a multiple sequence alignment and a motif investigation (Figures S1, S2 and S3 , respectively, available as Supplementary data at *Tree Physiology* Online, we could narrow down interesting AtLHT1 homologs from 13 to three candidates. The prediction of respective transmembrane domains (Figure S4 available as Supplementary data at *Tree Physiology* Online reduced the pool of candidates further to only two: PtLHT1.2 and PtLHT2. Finally, a phylogenetic analysis of all 10 AtLHT (Rentsch et al. 2007) and the 13 PtLHT proteins highlighted PtLHT1.2 as closest potential AtLHT1 homolog (Figure 1) and with this, corroborating results previously presented by Wu et al. 2015.

An in silico expression analysis of *PtLHT1.2* (Figure S11 available as Supplementary data at *Tree Physiology* Online (PopulusDB, www.popgenie.org; Yang et al. 2008, Sjodin et al. 2009, Sundell et al. 2015) showed that relative expression of *PtLHT1.2* was highest in expanded flowers and mature seeds from field samples, although the *PtLHT1.2* transcript was also present in leaves. This may reflect the involvement of this transporter in uptake of AAs in leaf mesophyll cells, similar to its Arabidopsis homolog AtLHT1 (Hirner et al. 2006). *PtLHT1.2* expression was also increased in infected leaves. This response has also been seen for *AtLHT1* (Liu et al. 2010, Farjad et al. 2018, Yoo et al. 2020). Thus, PtLHT1.2 may function in seed loading as well as leaf mesophyll transport and maintaining AA homeostasis during pathogen infection. To complement the in silico expression data, we next verified differential expression among different tissues of *PtrLHT1.2* in hybrid aspen (Figure S5 available as Supplementary data at *Tree Physiology* Online, Figure 2). In young seedlings, *PtrLHT1.2* was mostly expressed in old and middle-aged leaves. Expression was also found in fine and main roots as well as in stem and in younger leaves. Besides leaf mesophyll uptake, AtLHT1 has an important role in the uptake of AAs from the soil solution (Hirner et al. 2006, Svennerstam et al. 2007, Ganeteg et al. 2017). *PtrLHT1.2* root expression indicates that PtrLHT1.2 might be involved in the root uptake of AAs or in phloem and/or xylem loading of AAs for translocation.

Cloning and sequencing of *PtrLHT1.2* enabled a closer analysis of the protein. A sequence identity of 96.2% between PtLHT1.2 and PtrLHT1.2 falls into the expected, previously described, range (Sterky et al. 2004). Sequence dissimilarities between AtLHT1 and other known AtLHT1 homologs such as OsLHT1 (Guo et al. 2020) or LjLHT1.2 (Guether et al. 2011) range from 104 to 121 residues, respectively, and exceed the dissimilarities found between AtLHT1 and PtrLHT1.2. Hence, the confirmation of a high identity in protein sequence, motif arrangement and TMD structure between PtLHT1.2 and PtrLHT1.2 (Figure S6 available as Supplementary data at *Tree Physiology* Online, suggests that the isolated gene is a transmembrane protein. Crystal structures for plant AA transporters are largely missing, whereas plant nitrate transporters have been crystalized successfully (Parker and Newstead 2014). Due to the absence of structural data, the tertiary structure of PtrLHT1.2 was predicted, and the presence of pore-forming AAs confirmed that the protein acts as a solute transport protein (Figure S7 available as Supplementary data at *Tree Physiology* Online.

In order to analyze where the transporter is functional, the subcellular localization of a PtrLHT1.2-GFP fusion was determined in tobacco epidermal leaf cells (Figure 3). PtrLHT1.2 was found at the PM and in the ER. Such dual localization patterns, where transporter proteins are present in both cytosolic compartments and the cellular periphery, have been reported in many eukaryotic organisms. Examples for this are the human DIVALENT METAL TRANSPORTER1 (Tabuchi et al. 2002), the AMT ammonium transporters from *Dictyostelium discoideum* (Kirsten et al. 2008), the Arabidopsis iron transporters iron-regulated transporter1 (Barberon et al. 2011, Ivanov et al. 2014) and the oligopeptide transporter3 (Mendoza-Cózatl et al. 2014).

In the case of PtrLHT1.2, the strong ER localization suggests the existence of an intracellular transporter pool that can be rapidly mobilized in case of high AA requirement. Potentially, PtrLHT1.2 resides at the ER membrane during the absence of an interaction partner, which would initiate translocation to the PM upon a stress signal, possibly via a posttranslational modification. This hypothesis would be supported by the finding of a high molecular-weight form of PtrLHT1.2 (Figure S8 available as Supplementary data at *Tree Physiology* Online. Interestingly, AtLHT1 has been found to interact with a protein kinase in a membrane protein interactome study, supporting a potential protein modification (Chen et al. 2012). The Arabidopsis K^+^ channel AKT1 is regulated in a highly similar way. AtAKT1 is translocated from the ER to the PM through a synchronized interplay between the CBL4/CIPK6 module, which is strictly Ca^2+^-dependent (Held et al. 2011). PtrLHT1.2 transport along the secretory pathway, similar to the human AA transporter ATB^0,+^ (Kovalchuk et al. 2019), is further supported by the presence of several ER export motifs throughout the protein sequence (summary of plant ER export motifs in Hachez et al. 2013). Alternatively, PtrLHT1.2 might have an additional function to balance the cytoplasmic AA availability via export from the ER. Supporting such a possibility, the rice amino acid permease6 (OsAAP6), a regulator of grain protein content, was demonstrated to localize exclusively to the ER (Peng et al. 2014).

Applying this multi-step identification approach, *PtrLHT1.2* was identified as a promising candidate in our search for functional AA transporters in poplar.

### PtrLHT1.2 governs the uptake of neutral and acidic amino acids in hybrid aspen

An important step in the characterization of putative AA transporters is to verify their function as transporters and to determine substrate specificities and affinities. Functional analysis of AA transporters are usually performed in yeast or *Xenopus* oocyte expression systems (Hirner et al. 2006, Meyer et al. 2006). Using a yeast mutant, deficient in growth on L-Pro, L-citrulline and GABA, we proved that PtrLHT1.2 transports AAs (Figure S9 available as Supplementary data at *Tree Physiology* Online. The situation in the abovementioned systems, however, does not necessarily reflect the condition in planta. In Arabidopsis, AtLHT1 targets mainly neutral and acidic AAs (Hirner et al. 2006, Svennerstam et al. 2007,^2008^, ^2011^, Ganeteg et al. 2017). AtAAP5 fills the gap by transporting cationic AAs (Svennerstam et al. 2008, 2011). Therefore, we developed an in planta system for functional studies of PtrLHT1.2 by expressing the hybrid aspen gene in the Arabidopsis *lht1 aap5* double mutant. A constitutive expression of *PtrLHT1.2* was confirmed by RT-qPCR (Figure S10 available as Supplementary data at *Tree Physiology* Online. Both *PtrLHT1.2* expressing lines showed no signs of early senescence, caused by the loss of *AtLHT1* function (Hirner et al. 2006), hence resembling the WT phenotype (Figure 4A). This supports a functional role of PtrLHT1.2 in leaf mesophyll cells, consistent with its expression in this tissue (Figure S5 available as Supplementary data at *Tree Physiology* Online, Figure 2). When grown on 0.5 mM L-Gln as an N source, *PtrLHT1.2* expression not only reverted reduced N levels in seedlings, but also impaired plant growth, caused by the *lht1* mutation (Hirner et al. 2006, Svennerstam et al. 2007, Svennerstam et al. 2008). The expression even increased growth compared with WT, highlighting the potential of increased biomass production in future applications (Figure 4B and C). An additional promising finding, the increased affinity of *PtrLHT1.2* towards L-^14^C-Gln compared with L-^14^C-Arg (Figure 5A and B) confirmed our hypothesis that PtrLHT1.2 is an AA transporter, with similar AA affinities as AtLHT1 (Hirner et al. 2006, Svennerstam et al. 2007, 2008, 2011, Ganeteg et al. 2017). Analyzing the uptake potential of a range of different AAs by accessing the net uptake revealed a continuous transport of neutral and acidic AAs via PtrLHT1.2, as previously shown for AtLHT1 (Svennerstam et al. 2008) (Figure 5C). Lines *T4:4* and *T1:3* displayed not only a full rescue of the mutant uptake phenotype, but also distinct increased AA uptake compared with WT.

Organic N, such as AAs, may account for up to 80% of soil N supply in boreal forests (Inselsbacher and Näsholm 2012). Responsible for this might be an interplay between plant AA transporters and the colonization of roots by mycorrhizal fungi (Smith and Read 2008). It remains to be tested if there is a link between the mycorrhizal community and PtrLHT1.2 activity, as previously described for other AtLHT1 homologs (Guether et al. 2011, Zhao et al. 2014, Fochi et al. 2017). In addition, experiments substantiating the role of PtrLHT1.2 in hybrid aspen need to be addressed in the future. Our efforts in generating transgenic hybrid aspen RNAi lines targeting *PtrLHT1.2* did not yield in a significantly altered AA uptake profile. This can be explained by two poplar genome duplication events (Tuskan et al. 2006), suggesting that paralogous genes compensated for the reduced function of *PtrLHT1.2* in these lines. It can also not be ruled out that other *PtrLHT* family members hold similar physiology roles and could hence be the reason for the missing phenotype. Therefore, future work needs to be performed addressing the role of the other *PtrLHT* genes found in hybrid poplar.

However, the detailed characterization of a functional tree AA transporter in a heterologous system provides a first promising step in the understanding how trees take up AAs from the soil, which could have a big impact on future applications in forestry.

## Conclusions

Identification of specific proteins that contribute to increased N uptake and increased plant biomass is a valuable tool for breeding programs focusing on economic traits. Nitrogen transport is one of the major determinants for N-use efficiency (McAllister et al. 2012). In recent years, studies of AA transporters in the herbaceous plant Arabidopsis have provided crucial information for our understanding of AA transport. However, little is known about AA transporters in trees, and their importance for N-use efficiency in forest ecosystems. Thus, identification and characterization of such transporters, especially in economically valuable, short-rotation trees, is a critical step for N nutrition studies.

Our study presents the identification and characterization of a potential homolog of AtLHT1 in hybrid aspen and furthers our knowledge on tree AA transporters. The finding that PtrLHT1.2, upon overexpression, has the potential to increase biomass due to increased overall N uptake, can be used for targeted tree breeding programs as *Populus tremula* L. x *tremuloides* Michx. is a fast-growing hardwood in Northern Europe and can be used for the production of pulp and energy wood (Tullus et al. 2012). Breeding attempts of this species have already yielded clones with increased productivity and disease resistance, which highlights the potential of this hybrid aspen (Tullus et al. 2012).

Our study has hence provided a highly promising AA transporter candidate whose functional role in hybrid aspen and its potential for breeding and future cropping systems has to be addressed in future research efforts.

## Supplementary Material

Fig_S1_tpab029

Fig_S2_tpab029

Fig_S3_tpab029

Fig_S4_tpab029

Fig_S5_tpab029

Fig_S6_tpab029

FigureS7_tpab029

Fig_S8_tpab029

Fig_S9_tpab029

Fig_S10_tpab029

Fig_S11_tpab029

Table_S1_tpab029

Table_S2_tpab029

Table_S3_tpab029

Supplementary_Material_Legends_tpab029

## Data and materials availability

The novel *PtrLHT1.2* sequence is deposited in GenBank (http://www.ncbi.nlm.nih.gov), accession number MW590808. Plasmids or seeds that support the findings of this study are available from the corresponding author upon reasonable request.
