# Supplementary figures and images for: Organic nitrogen nutrition: LHT1.2 protein from hybrid aspen (Populus tremula L. x tremuloides Michx) is a functional amino acid transporter and a homolog of Arabidopsis LHT1
Source: Tree Physiol. 2021 Feb 25;41(8):1479–96. doi: 10.1093/treephys/tpab029 (PMC8359683; doi:10.1093/treephys/tpab029)

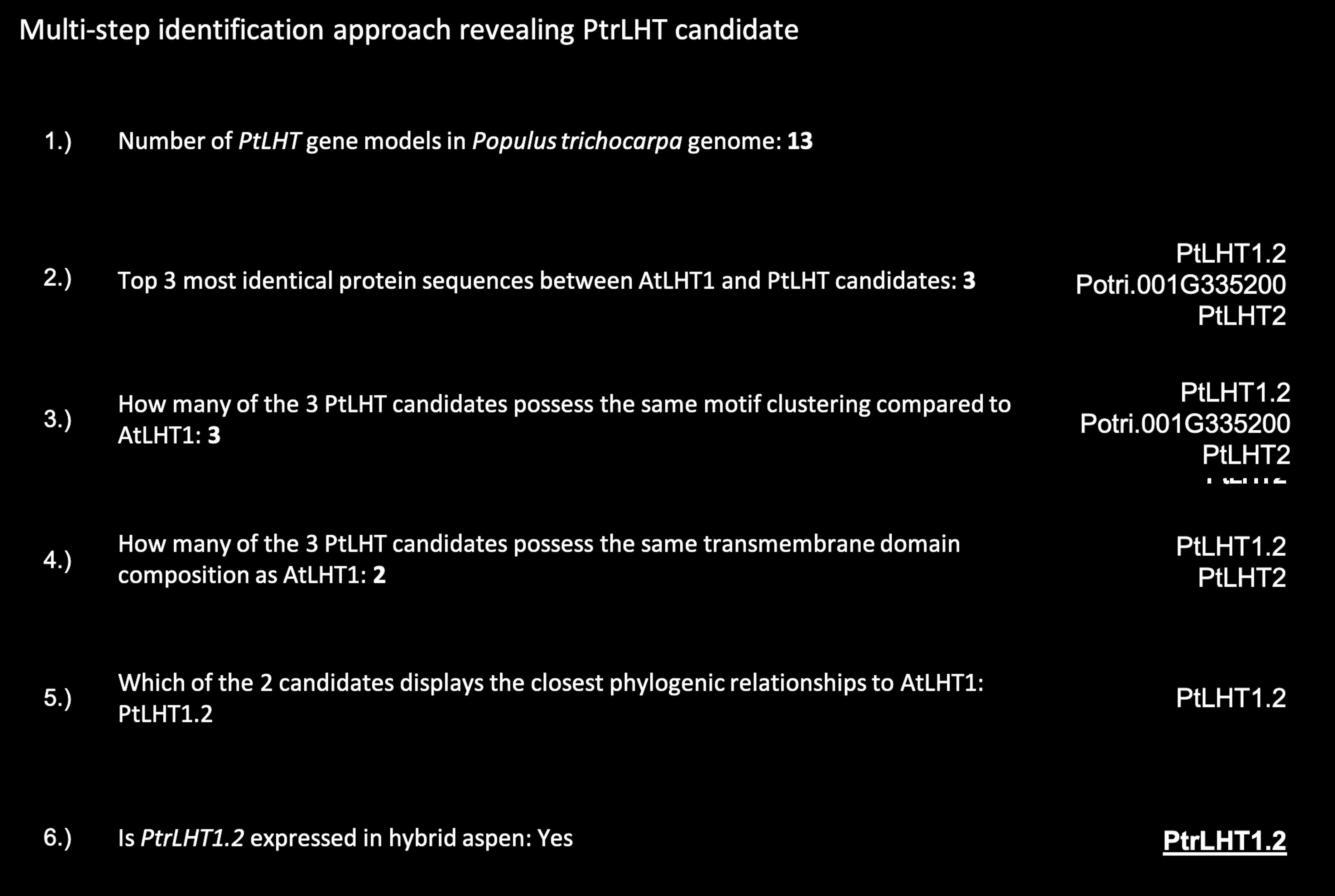

Supplement: Fig_S1_tpab029 [file fig_s1_tpab029.jpeg]

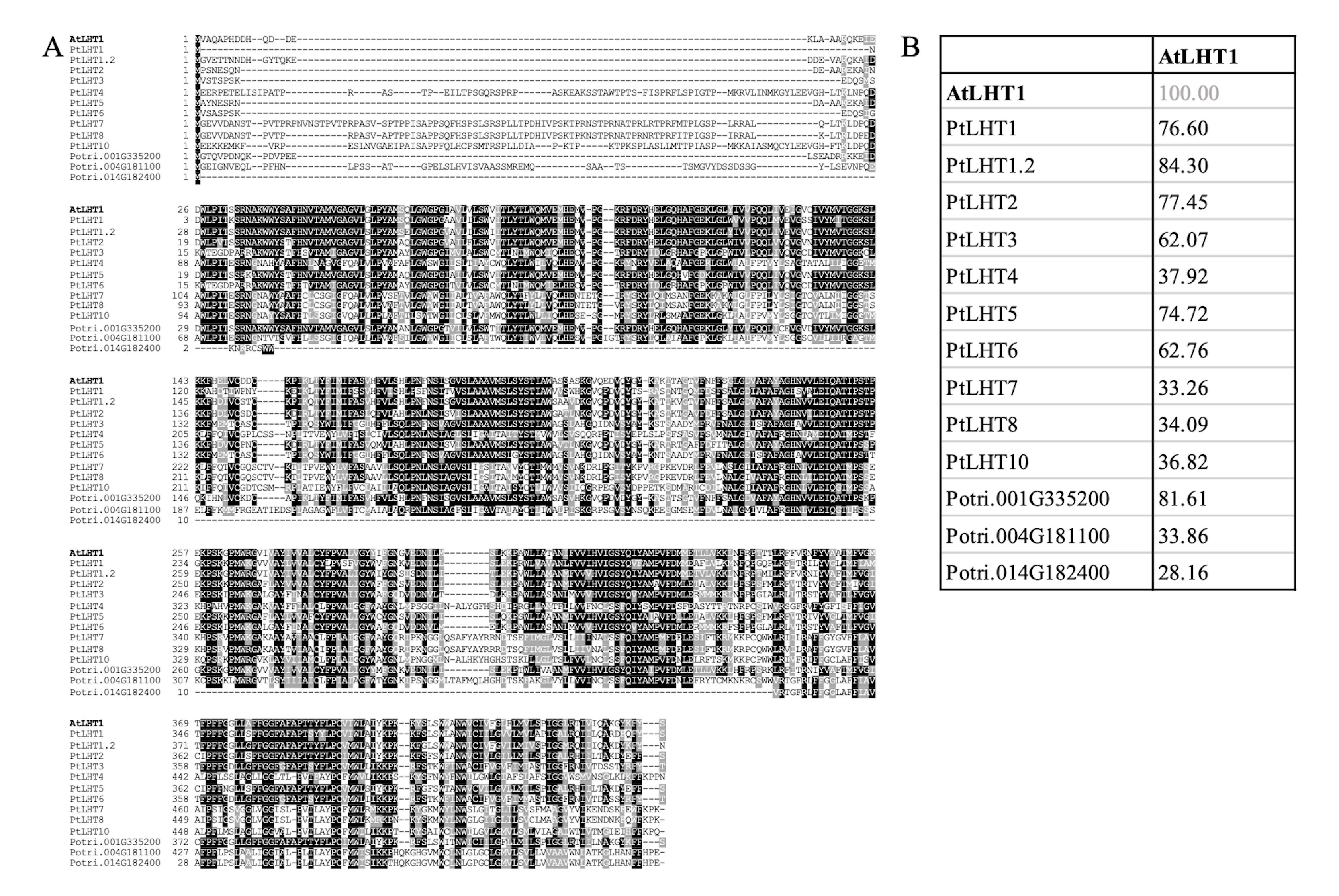

Supplement: Fig_S2_tpab029 [file fig_s2_tpab029.zip › Fig_S2_tpab029.png]

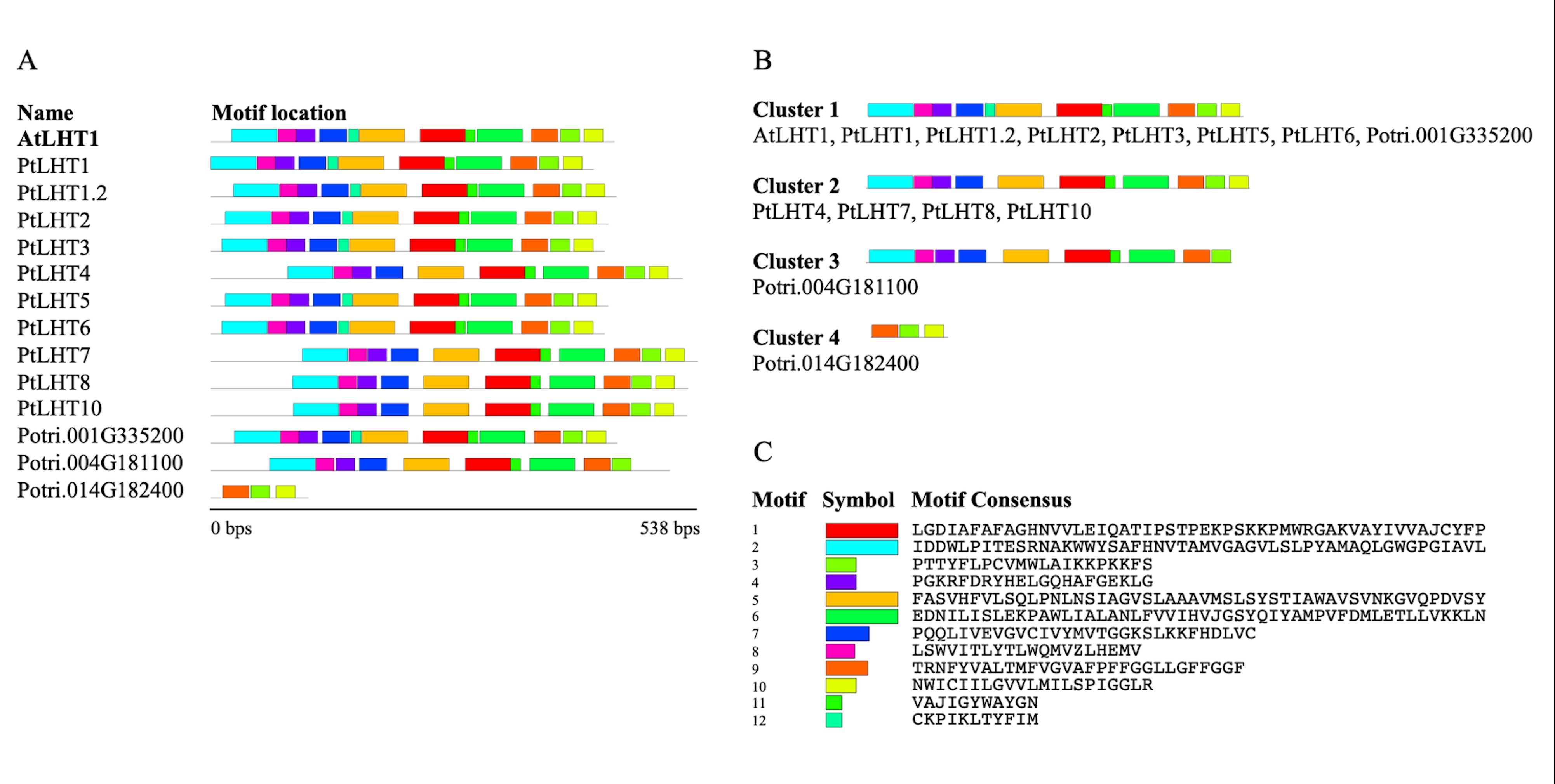

Supplement: Fig_S3_tpab029 [file fig_s3_tpab029.jpeg]

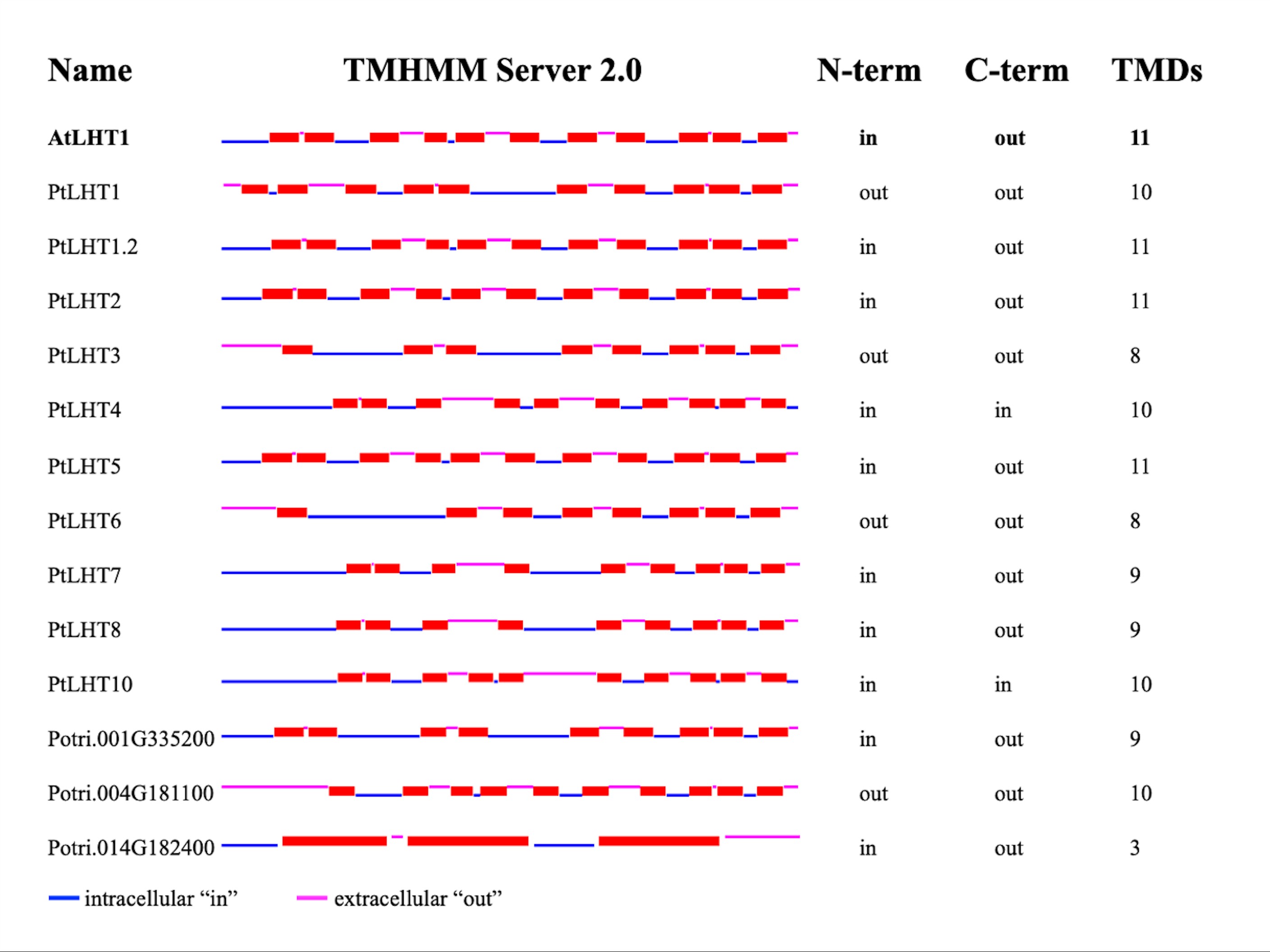

Supplement: Fig_S4_tpab029 [file fig_s4_tpab029.jpeg]

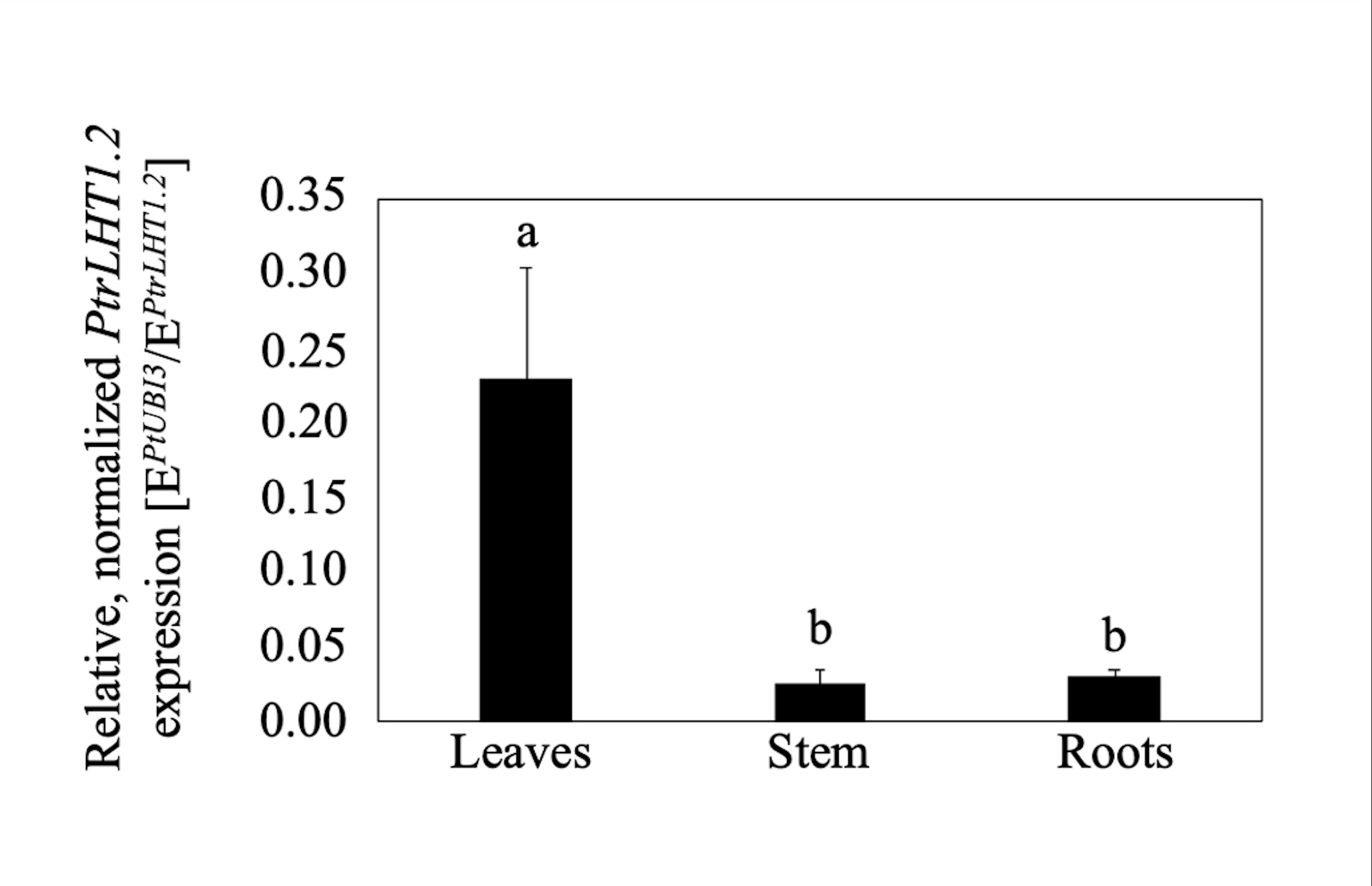

Supplement: Fig_S5_tpab029 [file fig_s5_tpab029.jpeg]

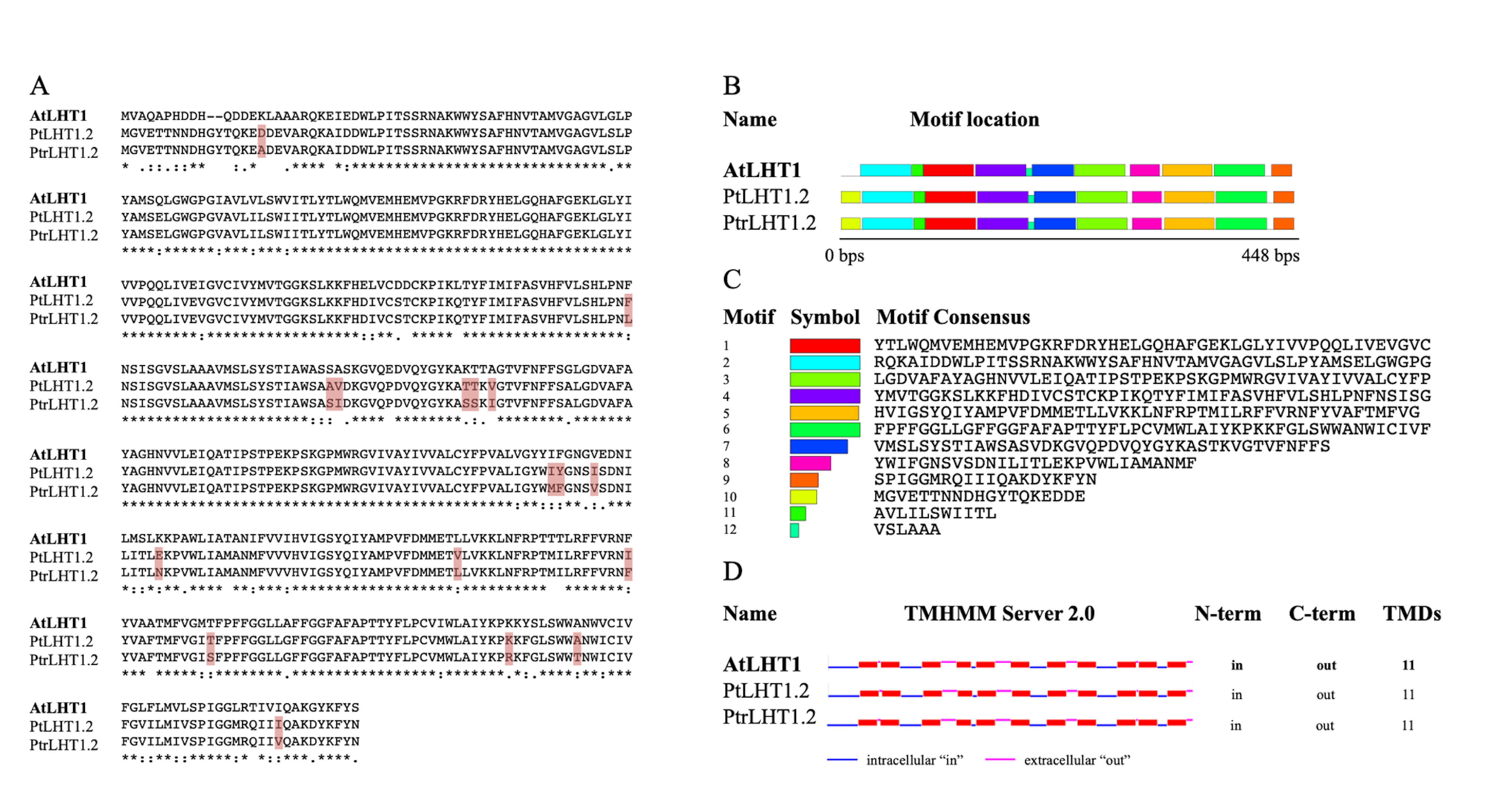

Supplement: Fig_S6_tpab029 [file fig_s6_tpab029.zip › Fig_S6_tpab029.png]

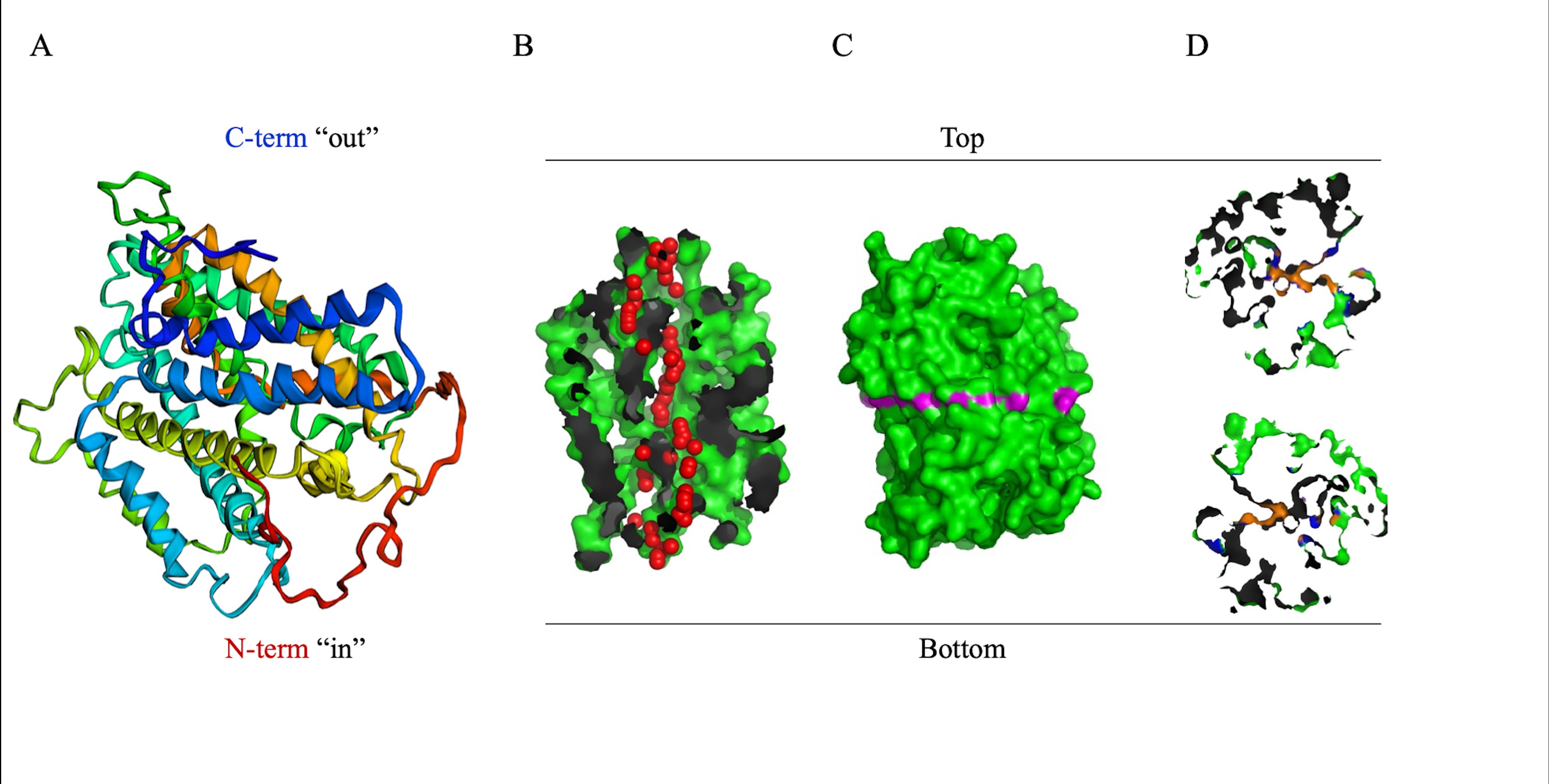

Supplement: FigureS7_tpab029 [file figures7_tpab029.jpeg]

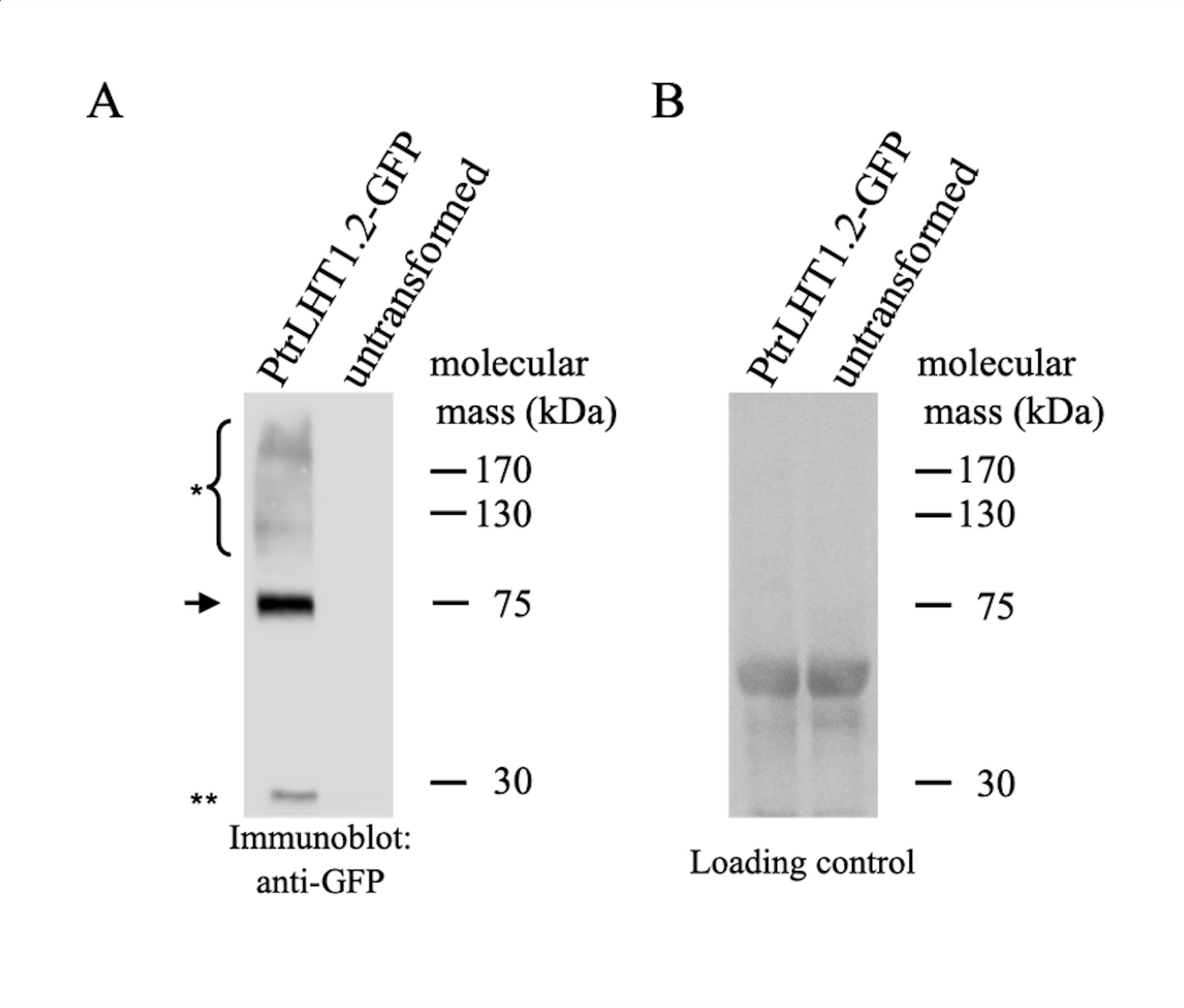

Supplement: Fig_S8_tpab029 [file fig_s8_tpab029.jpeg]

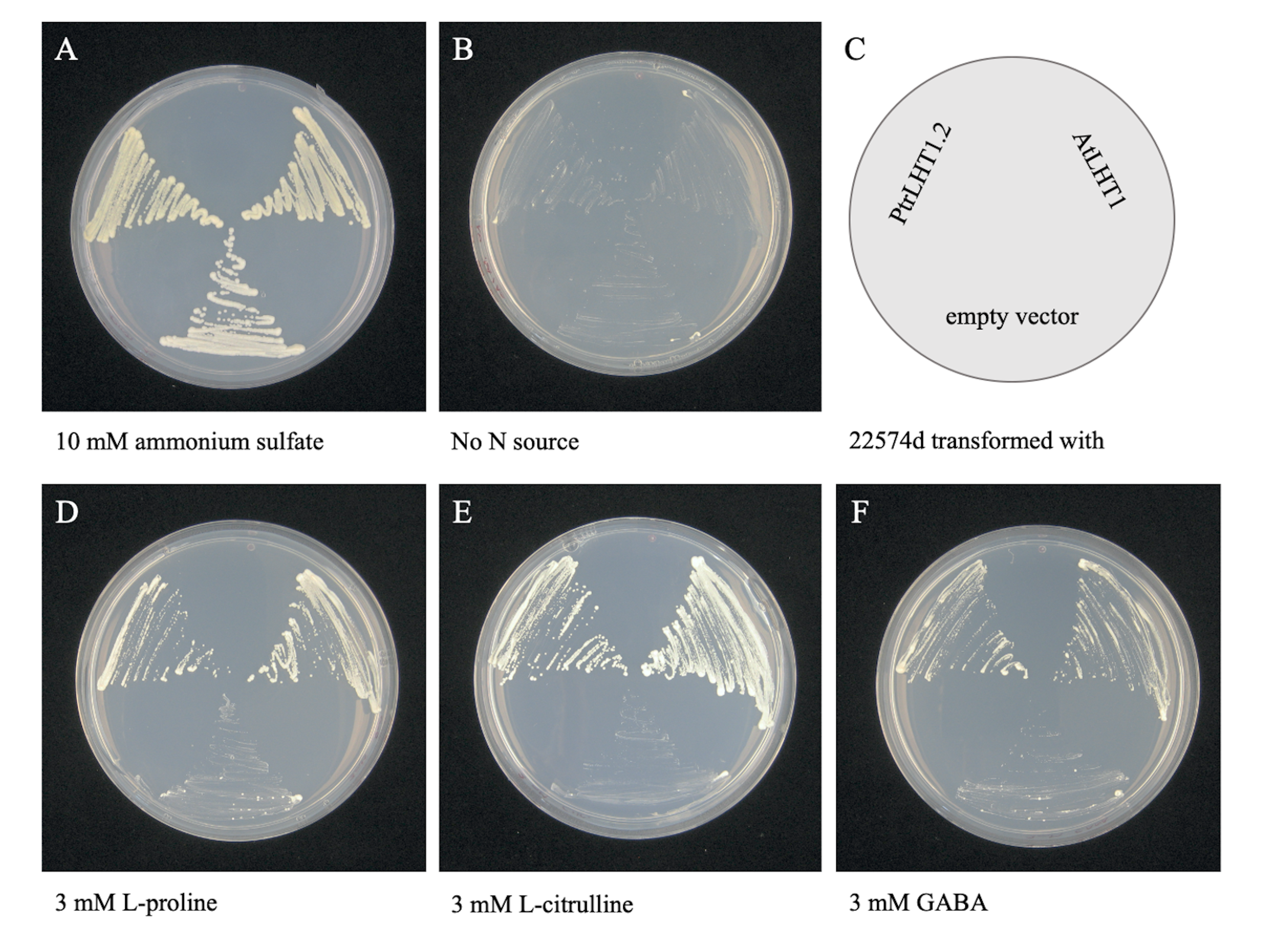

Supplement: Fig_S9_tpab029 [file fig_s9_tpab029.zip › Fig_S9_tpab029.png]

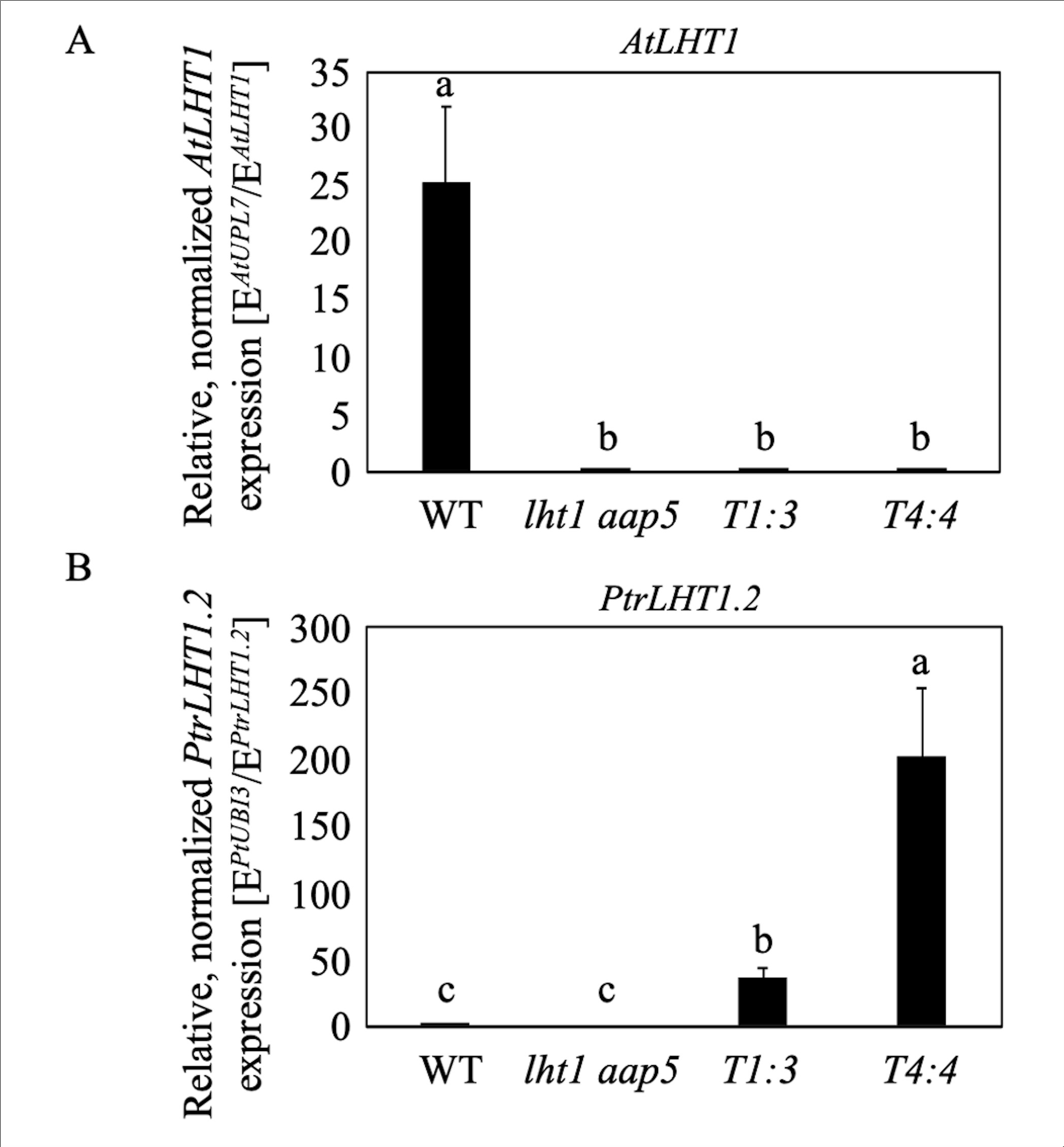

Supplement: Fig_S10_tpab029 [file fig_s10_tpab029.jpeg]

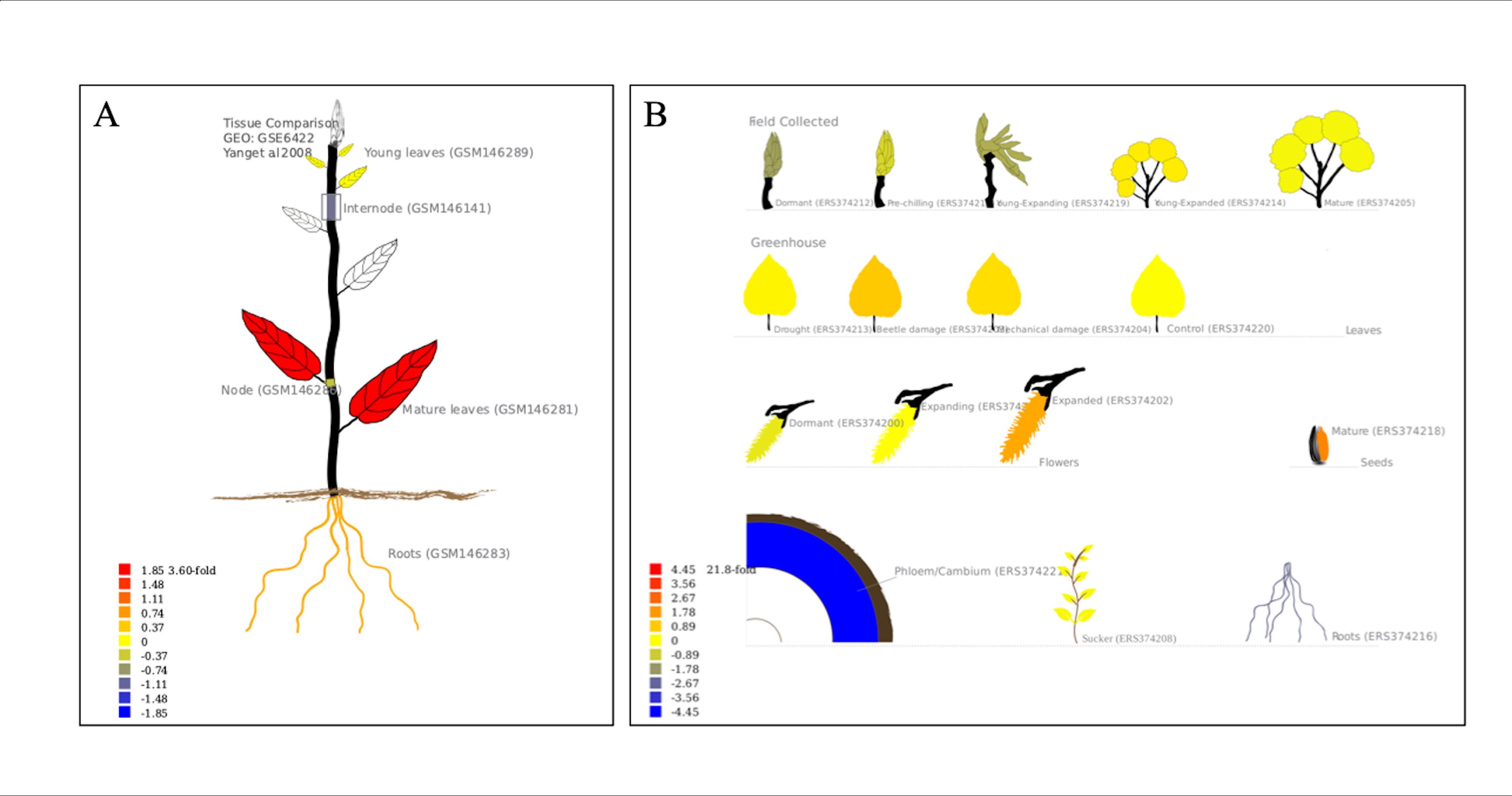

Supplement: Fig_S11_tpab029 [file fig_s11_tpab029.jpeg]
