# Supplementary material for: Organic nitrogen nutrition: LHT1.2 protein from hybrid aspen (Populus tremula L. x tremuloides Michx) is a functional amino acid transporter and a homolog of Arabidopsis LHT1
Source: Tree Physiol. 2021 Feb 25;41(8):1479–96. doi: 10.1093/treephys/tpab029 (PMC8359683; doi:10.1093/treephys/tpab029)
Supplement: Table_S1_tpab029 [file table_s1_tpab029.docx]

**Table S1:** Listed are thirteen *P. trichocarpa* *PtLHT* gene models, including their sequence ID according to Phytozome 12, their names by Wu et al. (2015), their names used in this study, the number of base pairs (bps), amino acids (AAs) as well as respective splice variants.

| **Sequence ID** | **Name Wu et al., 2015** | **Name this study** | **Number bps** | **Number AAs** | **Splice variants** |
| --- | --- | --- | --- | --- | --- |
| Potri.001G335200 | PtAAAP03 | Potri.001G335200 | 2670 | 449 | 0 |
| Potri.001G335300.1 | PtAAAP04 | PtLHT1.2 | 4796 | 448 | 4 |
| Potri.002G012900 | PtAAAP08 | PtLHT10 | 2366 | 526 | 0 |
| Potri.004G181100 | PtAAAP21 | Potri.004G181100 | 2451 | 507 | 0 |
| Potri.004G181200 | PtAAAP22 | PtLHT8 | 2838 | 527 | 0 |
| Potri.008G118000 | PtAAAP41 | PtLHT6 | 3292 | 435 | 0 |
| Potri.008G179000 | PtAAAP42 | PtLHT5 | 2098 | 439 | 0 |
| Potri.009G140800.1 | PtAAAP46 | PtLHT7 | 3202 | 538 | 2 |
| Potri.010G055800 | PtAAAP49 | PtLHT2 | 1965 | 439 | 0 |
| Potri.010G128300 | PtAAAP50 | PtLHT3 | 3866 | 435 | 0 |
| Potri.014G036500 | PtAAAP62 | PtLHT4 | 4382 | 521 | 0 |
| Potri.014G182400 | PtAAAP64 | Potri.014G182400 | 327 | 108 | 0 |
| Potri.015G091600 | PtAAAP65 | PtLHT1 | 2042 | 423 | 0 |
