# Supplementary material for: Organic nitrogen nutrition: LHT1.2 protein from hybrid aspen (Populus tremula L. x tremuloides Michx) is a functional amino acid transporter and a homolog of Arabidopsis LHT1
Source: Tree Physiol. 2021 Feb 25;41(8):1479–96. doi: 10.1093/treephys/tpab029 (PMC8359683; doi:10.1093/treephys/tpab029)
Supplement: Table_S2_tpab029 [file table_s2_tpab029.docx]

**Table S2:** List of primer sequences that were used for RT-qPCR and cloning. Primer efficiencies were noted for RT-qPCR primers.

| **Primer name** | **Fw Primer (5´-3´)** | **Rv Primer (5´-3´)** | **Efficiency** |
| --- | --- | --- | --- |
| RT-qPCR | | |  |
| PtUBI3_expression | CCAAGCCCAAGAAGATCAAGC | GCACCGCACTCAGCATTAGG | 1.9 |
| PtLHT1.2_expression | GCAGCACATGCAAACCGATCAA | CAGCCGACCAAGCAATCGTGGA | 1.9 |
| AtUPL7_expression | TTCAAATACTTGCAGCCAACCTT | CCCAAAGAGAGGTATCACAAGAGAC | 2.0 |
| AtLHT1_expression | GTTTGCCCCAACCACATACT | GGCGATAGGACCATCAAGAA | 1.9 |
| Cloning | | | |
| PtrLHT1.2_cloning | CACCGACATGGGTGTTGAAACT | TCAATTGTAGAACTTGTAGTCC | n/a |
| PtrLHT1.2_fusion | GGGGACAAGTTTGTACAAAAAAGCAGGCTATGGGTGTTGAAACTACCAAC | GGGGACCACTTTGTACAAGAAAGCTGGGTTATTGTAGAACTTGTAGTCCT | n/a |
| AtLHT1_cloning | GGGGACAAGTTTGTACAAAAAAGCAGGCTTAATGGTAGCTCAAGCTCCTCAT | GGGGACCACTTTGTACAAGAAAGCTGGGTCCTATGAGTAAAACTTGTATCC | n/a |
