# Supplementary material for: Organic nitrogen nutrition: LHT1.2 protein from hybrid aspen (Populus tremula L. x tremuloides Michx) is a functional amino acid transporter and a homolog of Arabidopsis LHT1
Source: Tree Physiol. 2021 Feb 25;41(8):1479–96. doi: 10.1093/treephys/tpab029 (PMC8359683; doi:10.1093/treephys/tpab029)
Supplement: Table_S3_tpab029 [file table_s3_tpab029.docx]

**Table S3:** Manders’ co-localization coefficients for PtrLHT1.2-marker combinations, given as mean and standard deviation.

| **Combination** | **Manders’ coefficients**  (M1 – fraction of green overlapping red,  M2 – fraction of red overlapping green) | **Sample number** |
| --- | --- | --- |
| PtrLHT1.2-GFP  OFP-HDEL | M_1_= 0.636 ± 0.031  M_2_= 0.732 ± 0.084 | 16 |
| PtrLHT1.2-GFP  AHA1-mRFP (whole-cell) | M_1_= 0.220 ± 0.023  M_2_= 0.624 ± 0.078 | 12 |
| PtrLHT1.2-GFP  AHA1-mRFP (PM region) | M_1_= 0.317 ± 0.027  M_2_= 0.582 ± 0.042 | 10 |
| PtrLHT1.2-GFP  AHA1-mRFP (plasmolysis) | M_1_= 0.343 ± 0.037  M_2_= 0.628 ± 0.056 | 06 |
