# Supplementary material for: Organic nitrogen nutrition: LHT1.2 protein from hybrid aspen (Populus tremula L. x tremuloides Michx) is a functional amino acid transporter and a homolog of Arabidopsis LHT1
Source: Tree Physiol. 2021 Feb 25;41(8):1479–96. doi: 10.1093/treephys/tpab029 (PMC8359683; doi:10.1093/treephys/tpab029)
Supplement: Supplementary_Material_Legends_tpab029 [file supplementary_material_legends_tpab029.docx]

**Supplementary data**

**Fig. S1:** Schematic summary of the applied steps leading to the identification of PtrLHT1.2. Thirteen PtLHT gene models are present in *Populus trichocarpa*. The number of potential AtLHT1 homologs was narrowed down from thirteen to three, by identifying the most identical protein sequences to AtLHT1 among the candidates. To further narrow down the number, sequence motives have been compared between the three candidates and AtLHT1. However, all three candidates displayed the same motive arrangement. In a next step, the composition of transmembrane domains was comparted between the three candidates and AtLHT1, which narrowed down the number to two potential poplar proteins. Further, the candidate was identified, which shared the closest phylogenetic relationship to AtLHT1, namely PtLHT1.2. In a last step expression of PtrLHT1.2 in hybrid aspen was confirmed.

**Fig. S2:** Multiple sequence alignment between Arabidopsis thaliana AtLHT1 and proposed LHT homologs in Populus trichocarpa (PtLHT). (A) Protein sequences of AtLHT1 (bold) and thirteen potential AA transporter encoding genes from P. trichocarpa were aligned based on the Clustal Omega algorithm. Black shadings represent conserved residues, grey shadings highlight similar AAs. (B) A Percent Identity Matrix highlights the degree of identity among all protein sequences (in %).

**Fig. S3:** Protein sequence characterization of AtLHT1 and thirteen potential poplar LHT homologs. (A) Conserved motives between the protein sequence of AtLHT1 (bold) and the thirteen PtLHT candidates are shown. Individual colors represent one particular motif. (B) Individual proteins are grouped into four different clusters, according to the similarity of the motif arrangements. (C) The consensus sequence of each motif is displayed.

**Fig. S4:** Prediction of transmembrane domain structure of AtLHT1 and thirteen potential poplar LHT homologs. TMHMM Server 2.0-based prediction of the orientation of the n-terminus and c-terminus of AtLHT1 (bold) and poplar LHT proteins. Additionally, the number of transmembrane domains (TMD) is shown, ranging from three to eleven.

**Fig. S5:** Relative *PtrLHT1.2* transcript levels in hydroponically grown hybrid aspen. Gene expression analysis of *PtrLHT1.2* in leaves, stems and roots of four-week-old hybrid aspen. The *Populus* ubiquitin extension protein gene *PtUBI3* was used for normalization. Data are represented as mean values ± SE. Different letters indicate statistically significant differences (p < 0.05, one-way ANOVA and Tukey post-hoc test, n=3).

**Fig. S6:** Characterization of PtrLHT1.2 protein sequence. (A) Multiple sequence alignment between the protein sequences of AtLHT1 (bold, top) and the potential poplar homologs PtLHT1.2 (middle) and PtrLHT1.2 (lower). AAs marked with red display differences in between the two Populus sequences. Asterisks represent identical AA residues in all three proteins. Highly similar and similar residues are represented by two dots and one dot, respectively. One representative alignment of 13 sequenced PtrLHT1.2 clones is shown. (B) Conserved motives between the protein sequence of AtlHT1 (bold), PtLHT1.2 and PtrLHT1.2 are shown. Individual colors represent one particular motif. (C) The consensus sequence of each motif is displayed. (D) The TMHMM Server 2.0 predicted the orientation of the n-terminus and c-terminus of AtLHT1 (bold), PtLHT1.2 and PtrLHT1.2. In all cases, an intercellular N-terminus and an extracellular C-terminus are suggested. Further, eleven transmembrane domains (TMDs) are suggested for all three proteins.

**Fig. S7:** Structural analysis of PtrLHT1.2. (A) Tertiary structure of PtrLHT1.2 was obtained from Phyre2 and presented with a chosen rainbow N- terminus (red) to C-terminus (blue) color structure. (B-D) Presentation of pore-forming structure within the PtrLHT1.2 protein structure. (B) Vertical section along the pore axis with highlighted pore centres (red) at 1 Ångström. (C) Horizontal section of the pore (magenta marking), presented as (D) top-view (top picture) or view from the bottom (lower picture) at 2 Ångström.

**Fig. S8:** Immunoblot analysis of tobacco-expressed PtrLHT1.2-GFP. (A) Immunoblot. Arrow indicates the predominant PtrLHT1.2-GFP form at 75 kDa. Asterisk indicates the high molecular-weight signal. Double asterisk indicates the potential free GFP at 27 kDa. (B): Loading control.

**Fig. S9:** Complementation of yeast AA uptake mutant by PtrLHT1.2 expression. Heterologous expression of PtrLHT1.2 in S. cerevisiae AA uptake mutant 22574d on different AAs as sole N source. (A) Yeast was transformed with either pDRf1::PtrLHT1.2, pDRf1::AtLHT1 (as positive control) or the empty vector (negative control) and tested on non-selective medium (10 mM ammonium sulfate). (B) Medium without N source served as negative control. (C) Schematic representation of yeast arrangement on petri dish. (D) Growth of yeast on 3 mM L-Pro, (E) 3 mM L-citrulline or (F) 3 mM GABA. Representative pictures were taken after a 10-day incubation at 30°C. The experiment was performed three times.

**Fig. S10:** Expression of *PtrLHT1.2* in Arabidopsis AA transporter mutants. Relative *PtrLHT1.2* transcript levels in root tissue of 19 days old WT, *lht1 aap5* double mutants and two individual lines of *lht1 aap5* double mutant plants expressing *PtrLHT1.2* (*T1:3* and *T4:4*). The Arabidopsis Ubiquitin Ligase gene *AtUPL7* was used to normalize the Arabidopsis RT-qPCR values. Bars represent mean values of a minimum of three biological replicates (n=3) ±SE.  Different letters represent statistical significance (one-way ANOVA and Tukey post-hoc test).

**Fig. S11:** *In silico* expression of *PtLHT1.2* in *Poplar trichocarpa* according to popgenie.org. (A) Visualization of microarray data ([Yang et al. 2008](#_ENREF_92)) displaying highest *PtLHT1.2* transcript abundance in mature poplar leaves. Data are deposited in the Gene Expression Omnibus (GEO) repository as data set GSE6422. (B) RNA-seq data by Sundell et al. 2015. Display of *PtLHT1.2* transcript abundance in different poplar tissues, with highest expression in expanded flowers and mature seeds.
